# Potato calcium sensor modules StCBL3-StCIPK7 and StCBL3-StCIPK24 negatively regulate plant immunity

**DOI:** 10.1186/s12870-023-04713-x

**Published:** 2024-01-05

**Authors:** Congcong Sun, Yuanyuan Li, Tingting Zhao, Weishuai Bi, Yingying Song, Xiangxiu Liang, Xiaodan Wang, Daolong Dou, Guangyuan Xu

**Affiliations:** 1 MOA Key Lab of Pest Monitoring and Green Management, Department of Plant Pathology, College of Plant Protection, China Agricultural University, Beijing, 100193 China; 2 College of Life Sciences, South China Agricultural University, Guangzhou, 510642 China; 3 College of Plant Protection, Nanjing Agricultural University, Nanjing, 210095 China

**Keywords:** *Phytophthora* resistance, Potato CBL-CIPK module, StCBL3, StCIPK7, StCIPK24

## Abstract

**Background:**

Potato late blight, caused by *Phytophthora infestans*, is the most devastating disease on potato. Dissecting critical immune components in potato will be supportive for engineering *P*. *infestans* resistance. Upon pathogens attack, plant Ca^2+^ signature is generated and decoded by an array of Ca^2+^ sensors, among which calcineurin B-like proteins (CBLs) coupled with plant specific CBL-interacting protein kinases (CIPKs) are much less explored in plant immunity.

**Results:**

In this study, we identified that two differential potato CBL-CIPK modules regulate plant defense responses against *Phytophthora* and ROS production, respectively. By deploying virus-induced gene silencing (VIGS) system-based pathogen inoculation assays, StCBL3 was shown to negatively regulate *Phytophthora* resistance. Consistently, StCBL3 was further found to negatively regulate PTI and ETI responses in *Nicotiana benthamiana*. Furthermore, StCIPK7 was identified to act together with StCBL3 to negatively regulate *Phytophthora* resistance. StCIPK7 physically interacts with StCBL3 and phosphorylates StCBL3 in a Ca^2+^-dependent manner. StCBL3 promotes StCIPK7 kinase activity. On the other hand, another StCBL3-interacting kinase StCIPK24 negatively modulating flg22-triggered accumulation of reactive oxygen species (ROS) by interacting with StRBOHB.

**Conclusions:**

Together, these findings demonstrate that the StCBL3-StCIPK7 complex negatively modulates *Phytophthora* resistance and StCBL3-StCIPK24 complex negatively regulate ROS production. Our results offer new insights into the roles of potato CBL-CIPK in plant immunity and provide valuable gene resources to engineer the disease resistance potato in the future.

**Supplementary Information:**

The online version contains supplementary material available at 10.1186/s12870-023-04713-x.

## Background

*Phytophthora infestans* is the most notorious pathogen on potato and tomato, causing late blight which is the most economically important potato disease [1]. To date, efficient control against late blight mainly rely on chemical control, which exerts potential threats to environment and human’s health. Therefore, green control technologies are urgent to develop for the pathogen management, among which genetic engineering method is the representative alternative for developing the durable and broad-spectrum resistance. Dissecting the plant immune signaling pathway and exploring novel immune-related components involved in potato defense to *P. infestans* is essential for mining the candidate gene resources to achieve the genetic improvement strategy. The plant immune system is composed of two layers: pattern-triggered immunity (PTI) and effector-triggered immunity (ETI), which have recently been implicated in close interplay for each other [2–5]. INF1, a protein with features of PAMPs from *P. infestans*, elicits host cell death and PTI responses [6]. To facilitate the infection, *P. infestans* secretes a battery of effectors into plant cell to interfere with PTI [7, 8]. However, effectors can be recognized by the cognate plant nucleotide-binding domain leucine-rich repeat (NLR) proteins and trigger ETI, accompanied by hypersensitive response (HR) [9]. Recent advances suggest that the PTI and ETI shared a series of common downstream signaling events, such as calcium influx and reactive oxygen species (ROS) production [10–12].

Serving as a ubiquitous second messenger, calcium plays an essential role in plant immunity. Upon immune activation, calcium influx mediated by calcium channels from apoplast into cytoplasm activates downstream signaling [13, 14]. In general, spatial and temporal dynamics are mainly decoded by an array of Ca^2+^ sensors, including conserved calmodulins (CaM), plant-specific calmodulin-like proteins, calcium dependent protein kinases (CDPKs or CPKs), calcineurin B-like proteins (CBLs) coupled with CBL-interacting protein kinases [15, 16]. CBLs and CIPKs constitute a signaling module in response to a variety of extracellular cues. Although the identities and roles of the CBL-CIPK network function in abiotic stress have well been studied [17], several studies have indicated that certain CIPKs or CBL-CIPK modules function in response to pathogen infection. For instance, CIPK6 negatively regulates PTI and ETI in *Arabidopsis* [18]. Similarly, CIPK14 also functions as a negative regulator of plant immune responses to *Pseudomonas syringae* in *Arabidopsis* [19]. Furthermore, the role of several CBL-CIPK modules in plant immunity has been studied in detail. For example, tomato SlCBL10-SlCIPK6 module are required for multiple effectors-NLRs-induced cell death [20]. Wheat TaCBL4 and its interacting partner TaCIPK8 positively regulate *Puccinia striiformis* f. sp. *tritici* (*Pst*) resistance [21]. In addition, wheat TaCIPK10 interacts and phosphorylates NH2, homologous to *Arabidopsis* NPR3/4, to regulate *Pst* resistance [22]. Overall, the roles of potato CBL-CIPK module in plant immunity are still scarce. Whether and how the CBL-CIPK network play roles in the defense to *P. infestans* is waiting to be revealed.

Besides calcium influx, NADPH oxidases RBOH-catalyzed extracellular ROS generation has been considered to be an important output for defenses responses. *Arabidopsis* contains 10 members of RBOHs, among which RBOHD plays key roles during plant-pathogen interactions [23, 24]. Accumulating evidence indicate fine-tuning regulation of RBOH by phosphorylation and ubiquitination is essential for ROS production. For instances, *Arabidopsis* receptor-like cytoplasmic kinase BIK1 and CPK5 directly interacts and differentially phosphorylates N terminus of RBOHD to contribute to PAMPs-induced ROS production, respectively [25–27]. Consistently, the potato StCDPK5 phosphorylates StRBOHB to boost ROS production upon *P. infestans* infection [28]. However, whether and how other calcium sensors monitoring StRBOHB’s activity is still unknown.

Here, we systemically investigate the immune roles of StCBLs in potato and identified StCBL3 negatively regulate disease resistance to *Phytophthora* infection. Moreover, overexpression of StCBL3 suppress diverse PAMPs-triggered immune responses and Avr3a-R3a-mediated cell death. Furthermore, our comprehensive analysis for StCIPKs identified StCIPK7 plays a negative role in defense to *Phytophthora* infection. A series of biochemical means verified that StCIPK7 physically interacts with StCBL3. In vitro phosphorylation assay displayed that StCIPK7 phosphorylates StCBL3 in Ca^2+^-dependent manner and StCBL3 promotes StCIPK7 kinase activity. We further demonstrated StCBL3-StCIPK7 acts as a functional module in regulating the disease resistance to *Phytophthora* infection. On the other hand, StCIPK24 contributes to flg22-triggered accumulation of ROS by interacting with StRBOHB. Collectively, our data demonstrate the StCBL3-StCIPK7 module negatively regulates *Phytophthora* infection and StCBL3-StCIPK24 regulates the ROS production via interacting StRBOHB. Our results offer new insights into the roles of potato CBL-CIPK in plant immunity and provide valuable gene resources to engineer the disease resistance potato in the future.

## Results

### StCBL3 plays a negative role in defense against *Phytophthora* infection

**Fig. 1:**
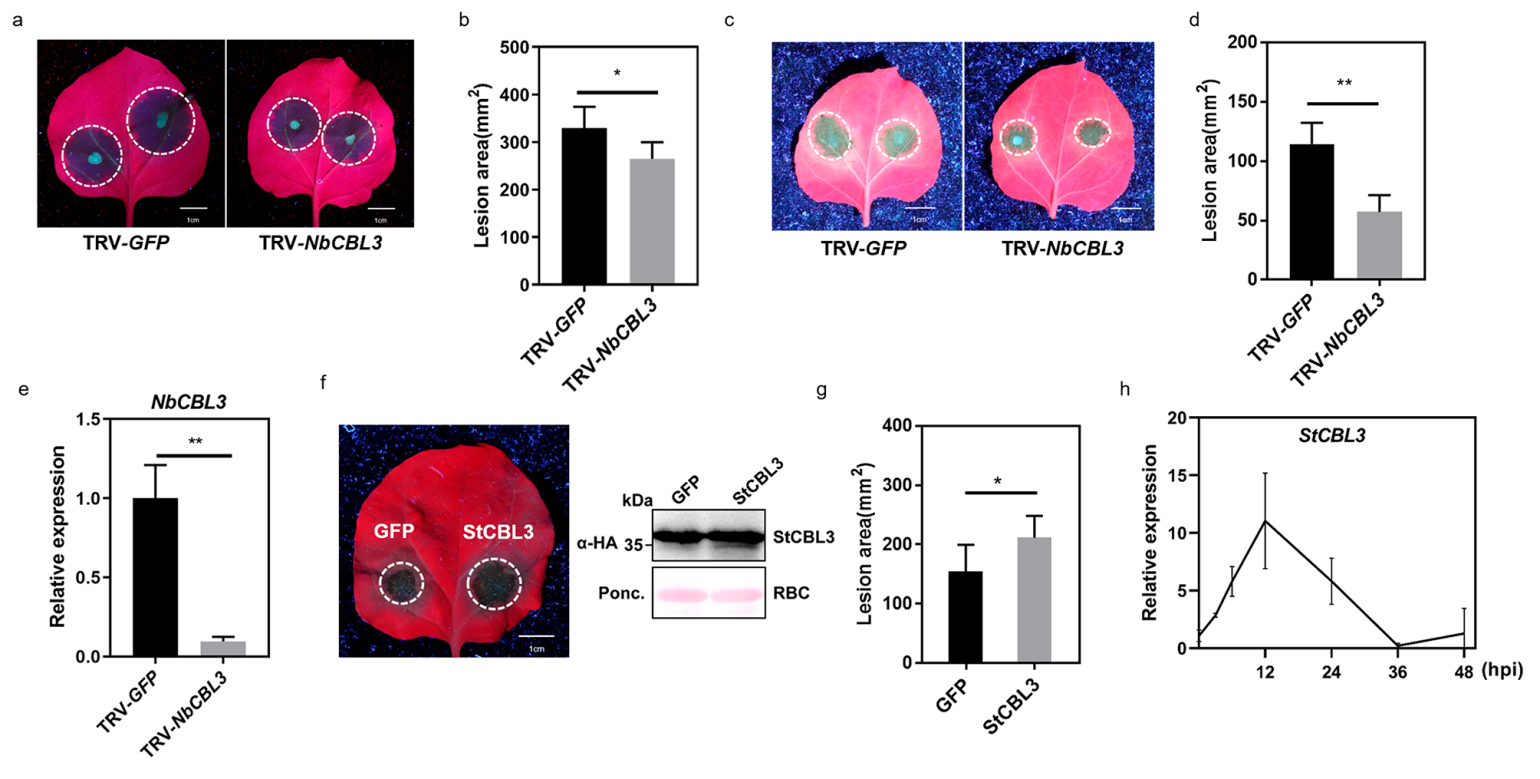
StCBL3 plays a negative role in defense against *Phytophthora* infection. (**a** and **b**) Silencing *NbCBL3* suppressed *P. capsici* infection compared with *GFP* control. Representative images of *P. capsici* lesions on TRV-*GFP* and TRV-*NbCBL3* silenced leaves. Leaf images were taken under UV light at 36 h post inoculation, scale bar = 1 cm (**a**). Meanwhile, lesion area was quantified by ImageJ (**b**). The data are shown as mean ± SD (n ≥ 6 leaves from different plants), and the asterisk indicates a significant difference with a Student’s *t*-test (*P < 0.05). The experiments were independently repeated 3 times with similar results. (**c** and **d**) Silencing *NbCBL3* suppressed *P. infestans* infection compared with *GFP* control. Representative images of *P. infestans* lesions on TRV-*GFP* and TRV-*NbCBL3* silenced leaves. Leaf images were taken under UV light at 6 dpi post inoculation, scale bar = 1 cm (**c**). Meanwhile, lesion area was quantified by ImageJ (**d**). The data are shown as mean ± SD (n ≥ 6 leaves from different plants), and the asterisk indicates a significant difference with a Student’s *t*-test (**P < 0.01). The experiments were independently repeated 3 times with similar results. (**e**) Silencing efficiency of *NbCBL3* was validated by RT-qPCR. *NbCBL3* expression was analyzed by RT-qPCR from *GFP* control and *NbCBL3*-silenced plants 2 weeks after VIGS. *NbActin* was used as an internal control. The data are shown as mean ± SD (**P < 0.01, Student’s *t*-test) from three independent repeats. (**f** and **g**) Transient expression of *StCBL3* in *N. benthamiana* leaves enhanced *P. infestans* colonization compared to half leaves inoculated with *Agrobacterium* expressing *GFP* empty vector. Leaf images were taken at 36 h post inoculation, scale bar = 1 cm (left panel of Fig. f). The GFP or StCBL3 proteins were immunoblotted with anti-HA antibody. Protein loading is indicated by Ponceau stain (Ponceau S) (right panel of Fig. f). Lesion area was quantified by ImageJ (**g**). The data are shown as mean ± SD (n ≥ 6 leaves from different plants), and the asterisk indicates a significant difference with a Student’s *t*-test (*P < 0.05). The experiments were independently repeated 3 times with similar results. (**h**) Relative expression levels of *StCBL3* were induced in response to *P. infestans* infection. Detached potato leaves were inoculated with *P. infestans*, and the samples were harvested at indicated time points. *StCBL3* expression was determined by RT-qPCR with the *StActin* gene as internal control. The data are shown as mean ± SD from three independent repeats

To identify the *CBL* genes in potato, 10 CBL protein sequences from *Arabidopsis* were used as queries to search the potato genome. A total of 13 putative CBLs were identified in the potato genome and designated as StCBL1 to StCBL13 according to their locations on chromosomes (Table 1). A phylogenetic tree of potato and *Arabidopsis* CBLs was constructed (Fig. S1a). To further explore whether StCBLs participate in the disease response to *Phytophthora* infection, we first checked the transcriptional patterns of these 13 *StCBL* genes in potato after infection by *P. infestans* using our in-house transcriptome data (Fig. S1b) [29]. We defined the *CBL* genes with 2-fold increase or decrease in potato after *P. infestans* infection as *P. infestans* responsive *CBL* genes, which include *StCBL3, StCBL6, StCBL10* and *StCBL11*. We further employed virus-induced gene silencing (VIGS) assays to analyze the roles of the potential StCBLs in response to *P. capsici* infection in *Nicotiana benthamiana*. Silencing *NbCBL3*, the close homolog of *StCBL3*, suppressed *P. capsici* infection, compared with the *GFP* control (Fig. 1a and b, S1c and S1d). Consistently, silencing *NbCBL3* in *N. benthamiana* also significantly reduced the lesion size caused by *P. infestans* infection, compared with the *GFP* control (Fig. 1c and e). On the other hand, compared with the *GFP* expression control, ectopic expression of *StCBL3* in *N. benthamiana* promotes *P. infestans* colonization (Fig. 1f and g). Therefore, these results indicate that StCBL3 plays a negative role in resistance to *Phytophthora* infections. Additionally, qPCR analyses showed that the transcript levels of *StCBL3* were significantly induced during the potato-*P. infestans* interaction and reached a peak level at 12 hpi (Fig. 1h), which is consistent with our transcriptome data.

**Table 1:** Characteristics of *StCBL* genes

Gene name	Gene ID	Chr.^1^	Genomic Location (bp)	CDS Length (bp)^2^	Protein Length (aa)^3^	pI^4^	MW (kDa)^5^
*StCBL1*	Soltu.DM.02G003810.1	2	15,398,622–15,399,296	675	224	4.63	25.90
*StCBL2*	Soltu.DM.03G022500.2	3	47,292,107–47,295,279	645	214	4.53	24.63
*StCBL3*	Soltu.DM.06G013800.1	6	38,805,408 − 38,801,973	645	214	4.65	24.62
*StCBL4*	Soltu.DM.06G018290.1	6	44,668,319–44,670,812	648	215	4.59	24.47
*StCBL5*	Soltu.DM.07G027610.1	7	56,697,477–56,704,208	660	219	4.74	25.22
*StCBL6*	Soltu.DM.08G001090.1	8	1,606,569–1,611,883	648	215	4.66	24.69
*StCBL7*	Soltu.DM.08G004770.1	8	5,991,908 − 5,988,428	642	213	4.82	24.64
*StCBL8*	Soltu.DM.08G007230.1	8	13,119,811–13,121,756	699	232	5.42	26.70
*StCBL9*	Soltu.DM.08G013470.1	8	39,563,588–39,570,325	774	257	4.64	29.88
*StCBL10*	Soltu.DM.08G023660.1	8	53,307,330 − 53,300,566	642	213	4.88	24.32
*StCBL11*	Soltu.DM.10G001000.1	10	834,491–840,443	669	222	4.89	25.52
*StCBL12*	Soltu.DM.12G008460.1	12	7,426,523 − 7,423,831	663	220	4.77	25.31
*StCBL13*	Soltu.DM.12G021010.1	12	49,717,521 − 49,708,742	675	224	4.76	25.68

^1^Chr., chromosome; ^2^CDS, coding sequence; ^3^aa, amino acids; ^4^pI, isoelectric point; ^5^MW, molecular mass

### StCBL3 negatively regulate multiple PAMPs-triggered immune responses and Avr3a/R3a-mediated cell death

**Fig. 2:**
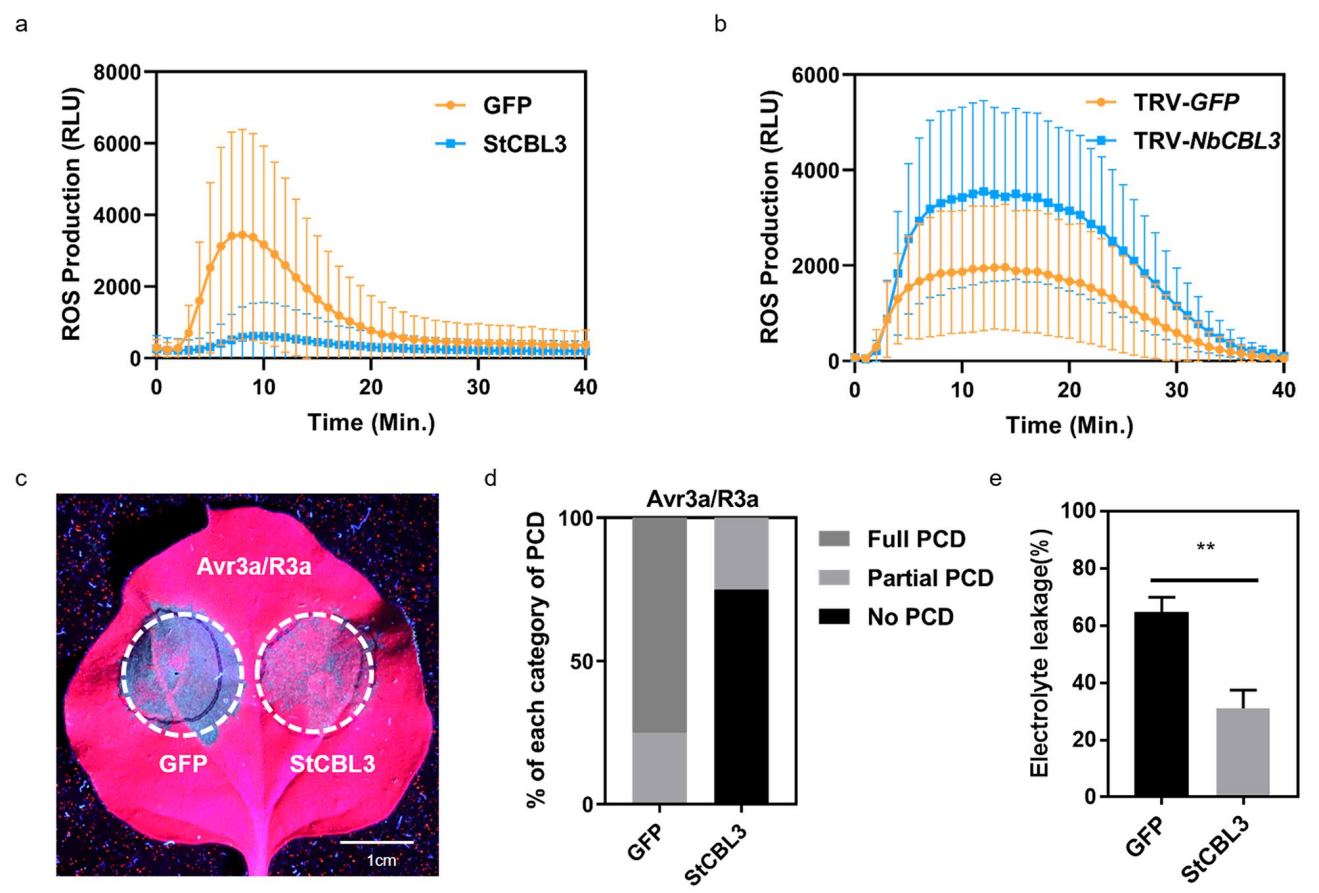
StCBL3 negatively regulates multiple PAMPs-triggered immune responses and Avr3a/R3a-mediated cell death. (**a**) Transient expression of *StCBL3* in *N. benthamiana* significantly inhibited flg22-triggered ROS production compared to the GFP control. Leaf discs were treated with 1 µM flg22 and ROS production was measured as relative light units (RLU) by a luminometer. The data are shown as mean ± SD (n ≥ 12 leaf discs). (**b**) Silencing *NbCBL3* enhanced flg22-induced ROS burst by nearly two-fold compared with the control TRV-*GFP* plants. Leaf discs were treated with 1 µM flg22 and ROS production was measured as relative light units (RLU) by a luminometer. The data are shown as mean ± SD (n ≥ 12 leaf discs). (**c** and **d**) Avr3a/R3a-induced cell death was dramatically inhibited by the expression of *StCBL3.* Cell death was visualized and photographed under UV light 48 h after infiltration, scale bar = 1 cm (**c**). The cell death extent was classified according to its extent: full PCD; partial PCD; no PCD (**d**). The experiments were independently repeated 3 times (each time with 6 biological replicates) with similar results. (**e**) Reduced electrolyte leakage measured in *N. benthamiana* plants were infiltrated with *Agrobacterium* cultures harboring *StCBL3* compared with *GFP*. The experiment was repeated three times with similar results. The data are shown as mean ± SD (n = 5 leaf discs), and the asterisk indicates a significant difference with a Student’s *t*-test (**P < 0.01)

To further dissect the roles of StCBL3 in PTI and ETI, we examined the involvement of StCBL3 in multiple PAMPs-triggered immune responses. PAMP-induced ROS production was analyzed on *StCBL3*-overexpressed or *NbCBL3*-silenced *N. benthamiana* plants. The results showed that transient overexpression of *StCBL3* significantly inhibited flg22-triggered ROS production, compared to the *GFP* control (Fig. 2a). Consistently, silencing the orthologue *NbCBL3* in *N. benthamiana* increased flg22-induced ROS burst by nearly two-fold, compared with the control TRV-*GFP* plants (Fig. 2b). In addition, we observed that overexpression of *StCBL3* also significantly inhibited the cell death induced by XEG1, a typical PAMP from *P. sojae* (Fig. S2a and S2b). However, overexpression of *StCBL3* did not affect the cell death triggered by INF1, another PAMP from *P. infestans* (Fig. S2c and S2d). These results indicated that StCBL3 negatively regulates multiple PAMPs-triggered immune responses. To further test whether StCBL3 is also involved in ETI, we tested the effect of *StCBL3* overexpression on the cell death induction by co-expression of the *P. infestans* effector Avr3a and its cognate NLR protein R3a in *N. benthamiana*. As shown in Fig. 2c and e, the Avr3a/R3a-induced cell death was dramatically suppressed by the expression of *StCBL3*. Taken together, the above results indicated that StCBL3 negatively regulate both PTI and ETI responses.

### Stable expression of *StCBL3* in *N. benthamiana* negatively regulates immune responses

**Fig. 3:**
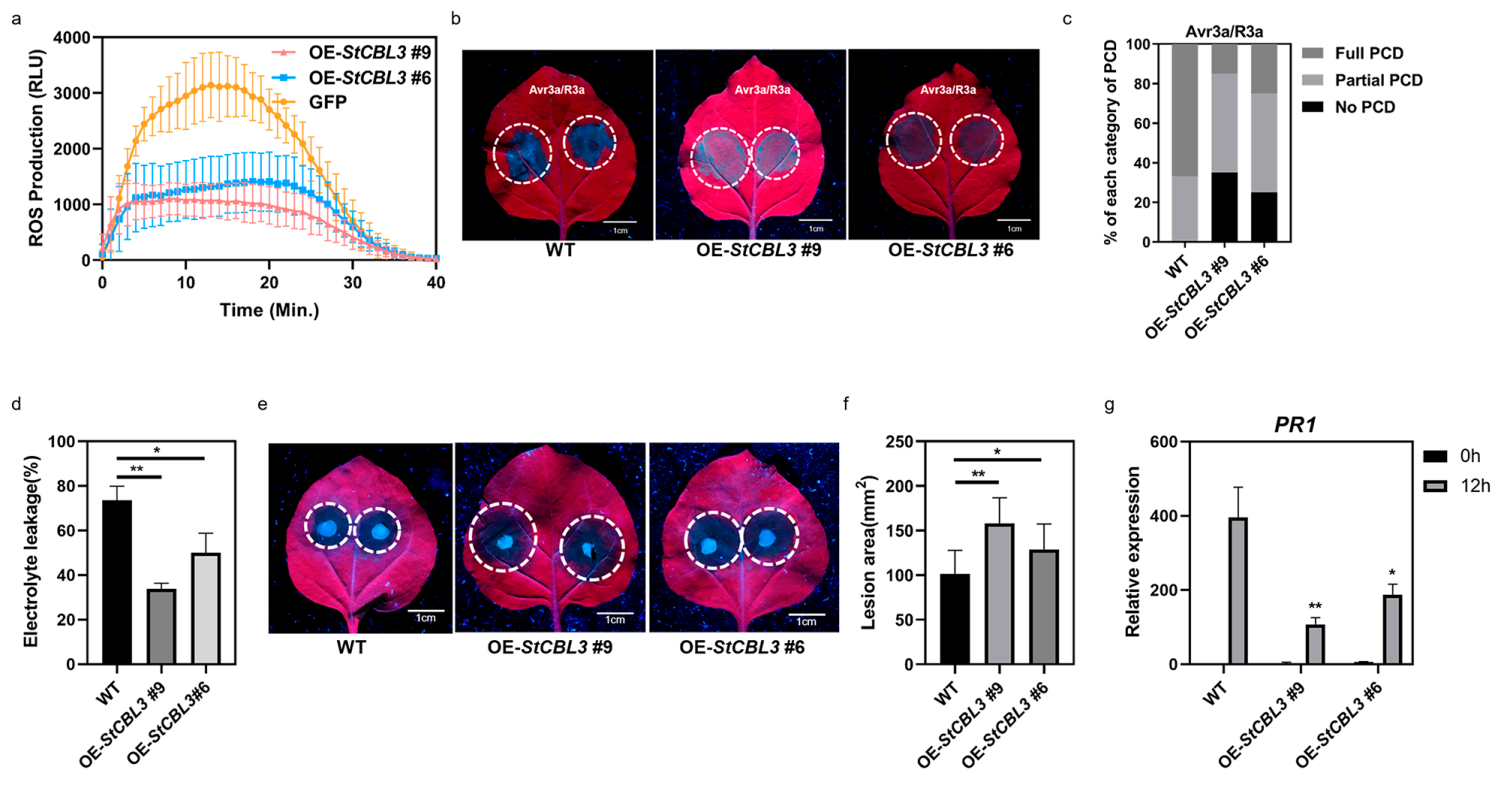
Stable expression of *StCBL3* in *N. benthamiana* negatively regulates immune responses. (**a**) Stable expression of *StCBL3* in *N. benthamiana* suppressed flg22-induced ROS production. Leaf discs from four-week-old soil-grown WT and two independent T3 homozygous lines (#6 and #9) plants were treated with 1µM flg22 and ROS production was measured as relative light units (RLU) by a luminometer. The data are shown as mean ± SD (n ≥ 12 leaf discs). (**b** and **c**) Attenuated cell death induced by Avr3a/R3a in stable *StCBL3*-overexpressed plants compared with wild-type plants (**b**). The cell death extent (**c**) was classified according to its extent: full PCD; partial PCD; no PCD. Two independent T3 homozygous lines (#6 and #9) were used. (**d**) Reduced electrolyte leakage measured in stable *StCBL3* transgenic plants compared with wild-type plants. Two transgenic lines (#6 and #9) were used. The experiment was repeated three times with similar results. The data are shown as mean ± SD (n = 5 leaf discs), and the asterisk indicates a significant difference with a Student’s *t*-test (*P < 0.05, **P < 0.01). (**e** and **f**) *StCBL3-*OE lines (#6 and #9) exhibited severer *P. capsici*-induced disease symptom and more lesion area compared with the wild-type plants. Leaf images were taken at 36 h post inoculation, scale bar = 1 cm (**e**). Lesion area was quantified by ImageJ (**f**). The data are shown as mean ± SD (n ≥ 6 leaves from different plants), and the asterisk indicates a significant difference with a Student’s *t*-test (*P < 0.05, **P < 0.01). The experiments were independently repeated 3 times with similar results. (**g**) Decreased expression levels of *NbPR1* in *StCBL3-*OE lines (#6 and #9) compared with wild-type plants after *P. capsici* treatment for 12 h. Transcription levels of *NbPR1* were determined by RT-qPCR with the *NbActin* gene as internal reference gene. The data are shown as mean ± SD and the asterisk indicates a significant difference with a Student’s *t*-test (*P < 0.05, **P < 0.01). The experiment was repeated three times

To further determine the role of StCBL3 in regulating plant immunity, we generated transgenic *N. benthamiana* plants (*StCBL3*-OE) overexpressing a HA-tagged StCBL3 under the control of the 35 S promoter. As shown in Fig. 3a, two independent homozygous *StCBL3*-OE transgenic lines were severely impaired in flg22-triggered ROS burst (Fig. 3a and S3). Additionally, *StCBL3*-OE lines exhibit less pronounced Avr3a/R3a-induced cell death (Fig. 3b and c), accompanied with less ion leakage (Fig. 3d). Upon inoculated with *P. capsici*, the *StCBL3*-OE lines exhibited severer disease symptoms and larger lesion areas compared with the wild-type plants (Fig. 3e and f). Moreover, the *P. capsici*-induced *PR1* gene expression was reduced in *StCBL3*-OE lines than in wild-type plants (Fig. 3g). Together, these results confirmed that StCBL3 negatively regulates plant immunity.

### StCIPK7 negatively regulates *Phytophthora* resistance

**Fig. 4:**
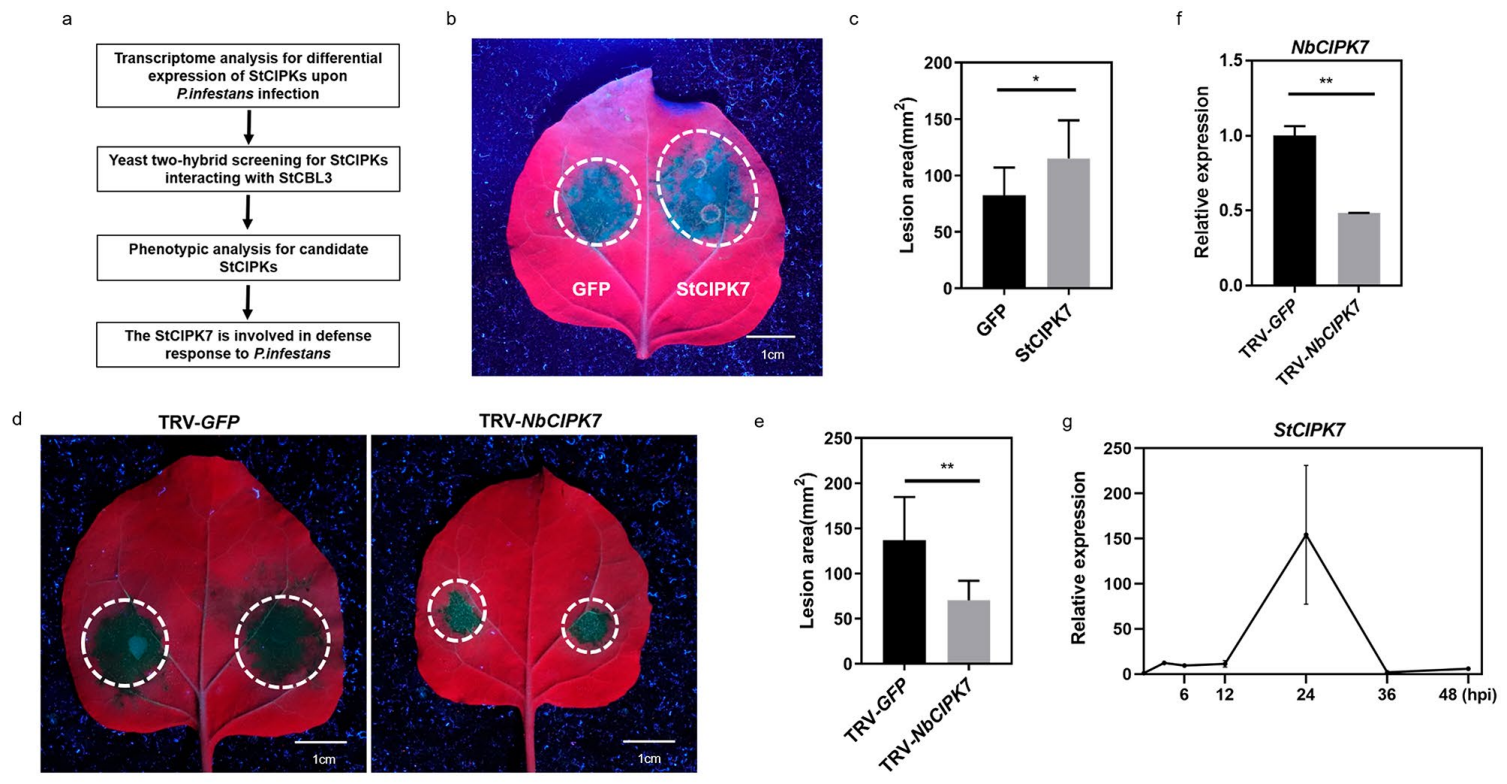
StCIPK7 negatively regulates resistance to *Phytophthora* infection. (**a**) Workflow to identify StCIPK7 involved in *Phytophthora* infection. (**b** and **c**) Transient expression of *StCIPK7* in *N. benthamiana* leaves enhanced *P. infestans* colonization compared to half leaves inoculated with *Agrobacterium* expressing *GFP* empty vector. Leaf images were taken at 36 h post inoculation, scale bar = 1 cm (**b**). Lesion area was quantified by ImageJ (**c**). The data are shown as mean ± SD (n ≥ 6 leaves from different plants), and the asterisk indicates a significant difference with a Student’s *t*-test (*P < 0.05). The experiments were independently repeated 3 times with similar results. (**d** and **e**) Silencing *NbCIPK7* suppressed *P. infestans* infection compared with *GFP* control. Representative images of *P. infestans* lesions on TRV-*GFP* and TRV-*NbCIPK7* silenced leaves. Leaf images were taken under UV light at 6 dpi post inoculation, scale bar = 1 cm (**d**). Meanwhile, lesion area was quantified by ImageJ (**e**). The data are shown as mean ± SD (n ≥ 6 leaves from different plants), and the asterisk indicates a significant difference with a Student’s *t*-test (**p < 0.01). The experiments were independently repeated 3 times with similar results. (**f**) Silencing efficiency of *NbCIPK7* was validated by RT-qPCR. *NbCIPK7* expression was analyzed by RT-qPCR from *GFP* control and *NbCIPK7*-silenced plants 2 weeks after VIGS. *NbActin* was used as an internal control. The data are shown as mean ± SD (**P < 0.01, Student’s *t*-test) from three independent repeats. (**g**) Relative expression levels of *StCIPK7* were induced in response to *P. infestans* infection. Detached potato leaves were inoculated with *P. infestans*, and the samples were harvested at indicated time points. *StCIPK7* expression was determined by RT-qPCR with the *StActin* gene as internal control. The data are shown as mean ± SD from three independent repeats

It has been documented that CBLs function together with CIPKs in mediating signaling transduction [17]. The potato genome contains 27 CIPKs [30] and a phylogenetic tree of potato and *Arabidopsis* CIPKs was constructed (Fig. S4a). We performed genetic and biochemical approaches to explore the potential CIPK paired with StCBL3 in plant defense to *Phytophthora* (Fig. 4a). First, based on the transcriptome analysis, we chose 13 differentially expressed StCIPKs as candidates, which displayed ≥ 2-fold increase or decrease in their transcripts after *P. infestans* infection (Fig. S4b). Then, we performed yeast-two-hybrid screening assays to test the interaction between StCBL3 and 13 StCIPKs. The result showed that 11 of the 13 CIPKs exhibit interaction with StCBL3 in yeast, including StCIPK7, 9, 11, 12, 15, 17, 19, 22, 23, 24, 27. StCIPK18 and StCIPK20 did not interact with StCBL3 in yeast (Fig. S4c). As shown in Fig. S4d and S4e, the split-luciferase complementation assays confirmed StCBL3 interacts with StCIPK1, 7, 9, 12, 15, 18, 22, 23, 24, but does not interact with StCIPK11, 17, 19, 20, 27. Finally, transient expression of 11 CIPKs in *N. benthamiana* followed by *P. infestans* inoculation assays revealed that overexpression of StCIPK7 promotes *P. infestans* infection (Fig. 4b and c, S4f and S4g).

To further confirm the involvement of StCIPK7 in *P. infestans* resistance, we employed VIGS to analyze the role of *NbCIPK7*, the homolog of *StCIPK7* in *N. benthamiana*, upon *P. infestans* infection. As shown in Fig. 4d and f, silencing *NbCIPK7* suppressed *P. infestans* infection compared with the *GFP* control in *N. benthamiana*. Therefore, these results indicate that, similar to StCBL3, StCIPK7 also negatively regulate plant resistance to *P. infestans* infection. Additionally, the expression of *StCIPK7* was induced in potato upon infection by *P. infestans* and reached a peak level at 24 hpi (Fig. 4g).

To further evaluate the role of StCIPK7 in plant immunity, we tested the involvement of StCIPK7 in ROS production triggered by flg22. As shown in Fig. S5a and S5b, neither transient overexpression of *StCIPK7* nor silencing *NbCIPK7* in *N. benthamiana* significantly affect the flg22-indcued ROS production, compared with the control. Additionally, transient overexpression of *StCIPK7* also did not have a pronounced effect on Avr3a/R3a-induced cell death in *N. benthamiana* (Fig. S5c and S5d). Overall, these results suggest that StCIPK7 plays a specific role in regulating the disease response to *Phytophthora* infection, not a role in PTI and ETI responses.

### StCBL3 interacts with StCIPK7

**Fig. 5:**
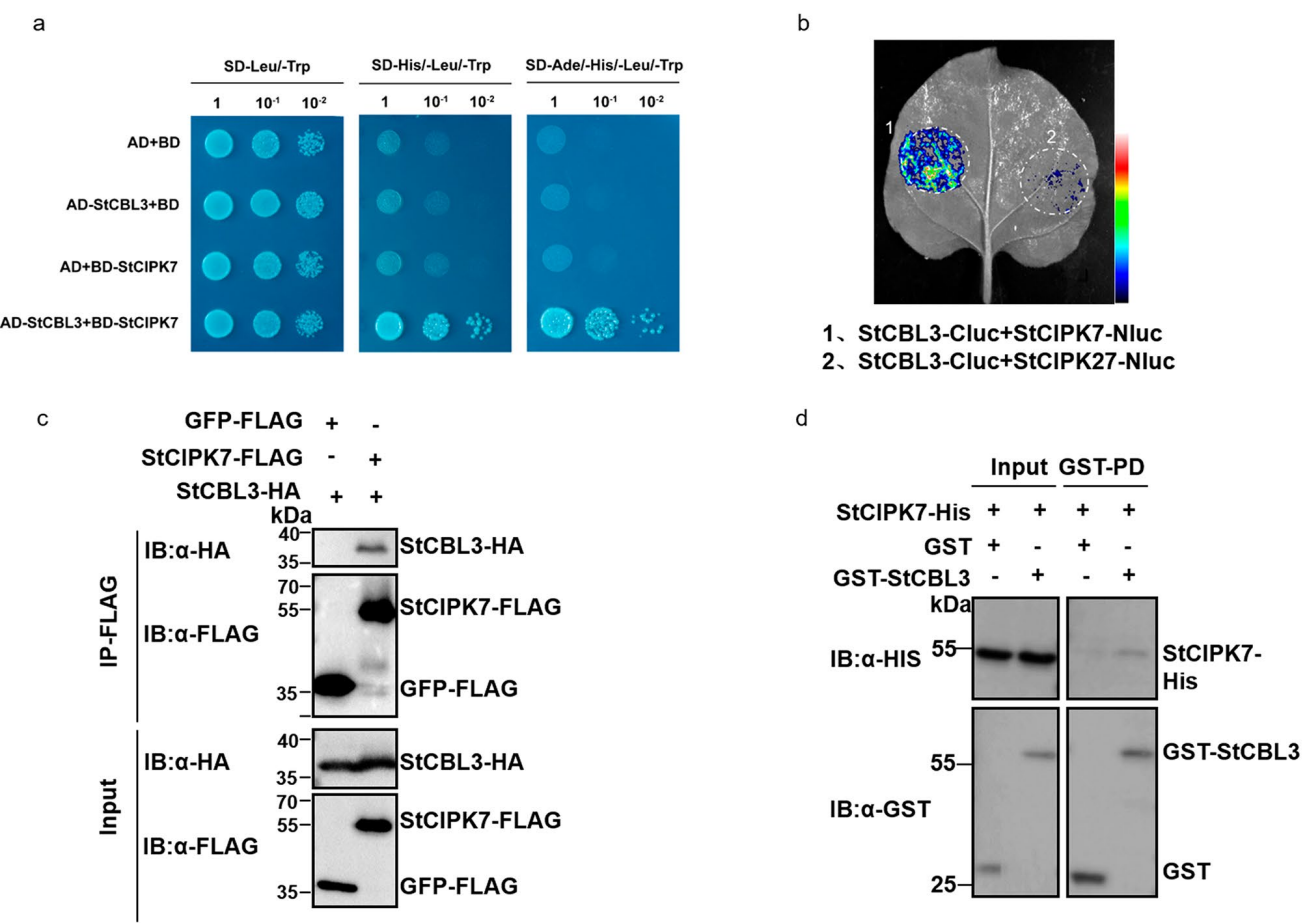
StCBL3 interacts with StCIPK7. (**a**) StCBL3 interacts with StCIPK7 in yeast. The interaction between StCBL3 and StCIPK7 was tested on synthetic defined medium without adenine, histidine, leucine and tryptophan and (SD-Ade-His-Leu-Trp). pGADT7 and pGBKT7 are empty vectors. Serial dilutions of the yeast colonies were plated. (**b**) StCBL3 associates with StCIPK7 in the split-luciferase assay. Constructs carrying StCBL3-Cluc and StCIPK7-Nluc were co-expressed in *N. benthamiana* leaves for 2 days. The infiltrated leaf was detached and treated with 1 mM luciferin, and the bioluminescence image was captured by a CCD camera. The StCIPK27 was used as a negative control. The pseudo-color bar shows the range of luminescence, indicating the interaction intensity. (**c**) The association between StCBL3 and StCIPK7 in *N. benthamiana* were detected by co-immunoprecipitation. StCBL3-HA was co-expressed with StCIPK7-FLAG or GFP-FLAG in *N. benthamiana* for 36 h. Total proteins were immunoprecipitated with an α-FLAG antibody (α-FLAG IP) and immunoblotted with a-HA (IB: α-HA) or α-FLAG (IB: α-FLAG; top two panels). Protein inputs are shown with immunoblotting before immunoprecipitation (bottom two panels). (**d**) StCBL3 interacts with StCIPK7 in an in vitro pull-down (PD) assay. GST or GST-StCBL3 immobilized on glutathione Sepharose beads was incubated with StCIPK7-His proteins. The beads were washed and pelleted for immunoblotting with anti-GST and anti-His antibody PD: GST; IB: α-HA; top panel). Protein inputs are shown with immunoblotting before immunoprecipitation (bottom two panels)

The yeast two-hybrid assay revealed StCBL3 interacts with StCIPK7 (Fig. 5a and Fig. S4c). To further confirm their interaction, a co-immunoprecipitation (coIP) assay with the co-expressed FLAG-tagged StCIPK7 and hemagglutinin (HA)-tagged StCBL3 in *N. benthamiana* leaves indicated that StCIPK7 associates with StCBL3 (Fig. 5b). Split-luciferase complementation assays in *N. benthamiana* confirmed that StCBL3 specifically interacted with StCIPK7, but not with StCIPK27 (Fig. 5c). In addition, the His-tagged StCIPK7 was pulled down by glutathione S-transferase (GST)-tagged StCBL3 but not GST itself in an in vitro pull-down assay (Fig. 5d). Together, these results demonstrated that StCBL3 interacts with StCIPK7 in vitro and in vivo.

### StCBL3 cooperates with StCIPK7 to regulate *Phytophthora* resistance

**Fig. 6:**
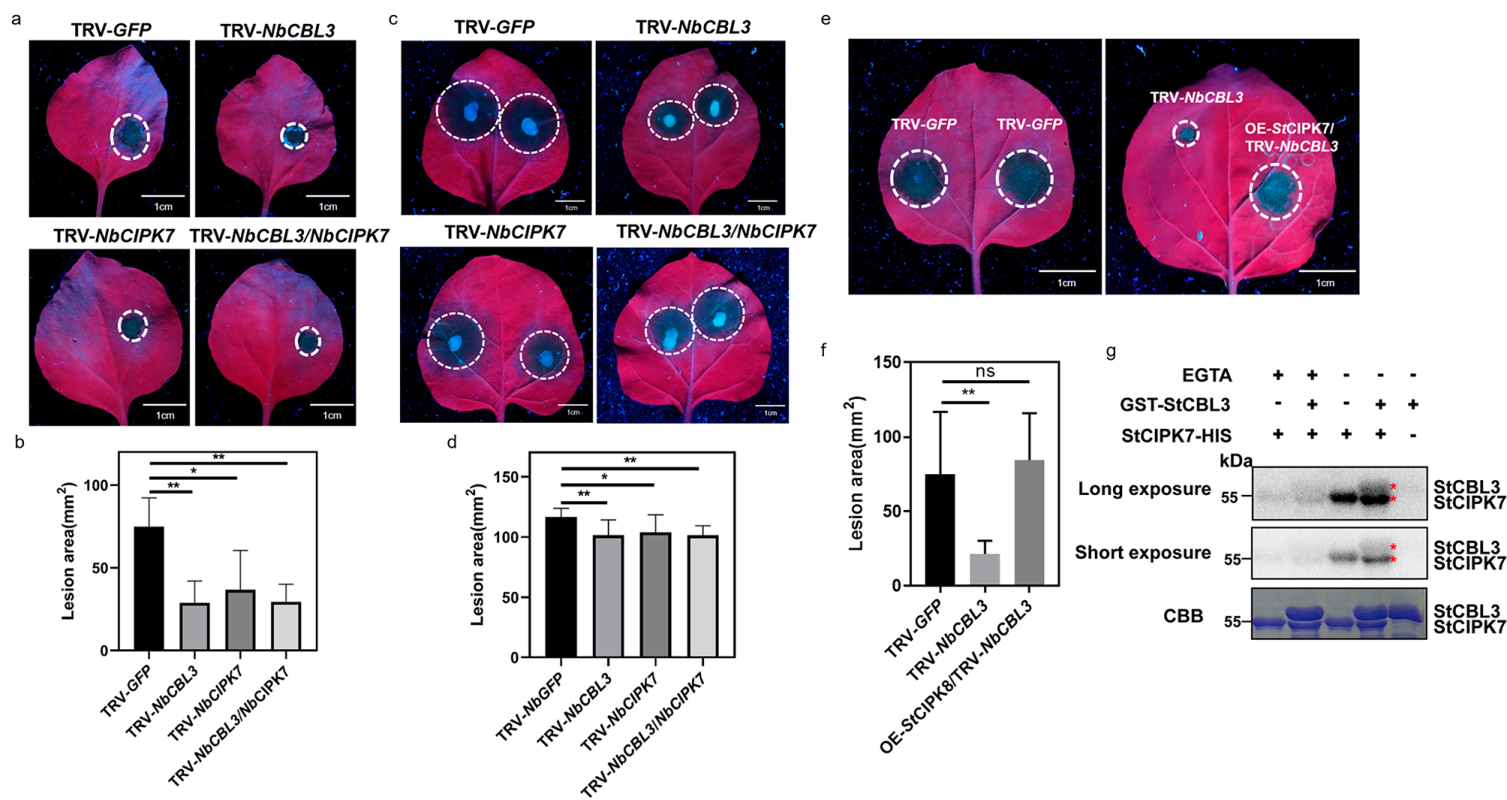
StCBL3 cooperates with StCIPK7 to regulate disease resistance to *Phytophthora* infection. (**a** and **b**) TRV-*NbCIPK7*/TRV-*NbCBL3* plants showed similar reduction in *P. infestans-*induced disease symptom and lesion area as single silenced TRV-*NbCIPK7* or TRV-*NbCBL3*. Representative images of *P. infestans* lesions on TRV-*GFP*, TRV-*NbCBL3*, TRV-*NbCIPK7* or TRV-*NbCIPK7*/TRV-*NbCBL3* silenced leaves. Leaf images were taken under UV light at 6 dpi post inoculation, scale bar = 1 cm (**a**). Meanwhile, lesion area was quantified by ImageJ (**b**). The data are shown as mean ± SD (n ≥ 6 leaves from different plants), and the asterisk indicates a significant difference with a Student’s *t*-test (*P < 0.05, **P < 0.01). The experiments were independently repeated 3 times with similar results. (**c** and **d**) TRV-*NbCIPK7*/TRV-*NbCBL3* plants showed similar reduction in *P. capsici-*induced disease symptom and lesion area as single silenced TRV-*NbCIPK7* or TRV-*NbCBL3*. Representative images of *P. capsici* lesions on TRV-*GFP*, TRV-*NbCBL3*, TRV-*NbCIPK7* or TRV-*NbCIPK7*/TV-*NbCBL3* silenced leaves. Leaf images were taken under UV light at 36 h post inoculation, scale bar = 1 cm (**c**). Meanwhile, lesion area was quantified by ImageJ (**d**). The data are shown as mean ± SD (n ≥ 6 leaves from different plants), and the asterisk indicates a significant difference with a Student’s *t*-test (*P < 0.05, **P < 0.01). The experiments were independently repeated 3 times with similar results. (**e** and **f**) The expression of *StCIPK7* restored *P. infestans-*induced disease symptom and lesion area reduction caused by silencing *NbCBL3* to the *GFP* control level. Representative images of *P. infestans* lesions on TRV-*GFP*, TRV-*NbCBL3*, OE-*StCIPK7*/TRV-*NbCBL3* leaves. Leaf images were taken under UV light at 6 days post inoculation, scale bar = 1 cm (**e**). Meanwhile, lesion area was quantified by ImageJ (**f**). The data are shown as mean ± SD (n ≥ 6 leaves from different plants), the asterisk indicates a significant difference with a Student’s *t*-test (**P < 0.01) and “ns” indicates no significant difference. The experiments were independently repeated 3 times with similar results. (**g**) The StCIPK7 kinase activity was enhanced by adding the StCBL3 protein and the phosphorylation of StCBL3 by StCIPK7 was blocked in the presence of Ca^2+^ chelator EGTA. The phosphorylation reactions were performed using GST-StCIPK7 as the kinase and StCBL3-His as the substrate. After separation by SDS-PAGE, the phosphorylated proteins were detected by immunoblotting by autoradiography (top two panels). The protein inputs were assessed by Coomassie Brilliant Blue staining (bottom panel), and the target protein bands are marked by asterisks

To further investigate whether StCBL3 and StCIPK7 regulate plant immunity through the same pathway, we silenced *NbCBL3* and *NbCIPK7* individually or together in *N. benthamiana* before we inoculated these plants with *P. infestans* and *P. capsici*, respectively. As shown in Fig. 6a and d, similar reductions in lesion area were observed upon silencing *NbCIPK7* and *NbCBL3* individually or together, compared with the TRV-*GFP* control, suggesting that CIPK7 and CBL3 genetically function in the same pathway. To further confirm the genetic interaction between StCBL3 and StCIPK7, we first silenced *NbCBL3*, then transiently overexpressed *StCIPK7* in *N. benthamiana* before inoculation with *P. infestans*. As shown in Fig. 6e and f, overexpression of *StCIPK7* blocked the lesion area reduction caused by *NbCBL3* silencing, indicating that StCIPK7 overaccumulation could complement the defects caused by partial loss of StCBL3. We further performed in vitro kinase assays to test the potential reciprocal regulation of StCBL3 and StCIPK7. StCIPK7 exhibited autophosphorylation activity and phosphorylated StCBL3 in vitro (Fig. 6g). Notably, the kinase activity of StCIPK7 was enhanced by adding the StCBL3 protein, and the phosphorylation of StCBL3 by StCIPK7 was blocked in the presence of Ca^2+^ chelator EGTA (Fig. 6g), indicating that, upon binding Ca^2+^, StCBL3 could further stimulate StCIPK7 activity to trigger downstream signaling.

### StCIPK24 associates with StRBOHB and negatively regulate flg22-induced ROS production

**Fig. 7:**
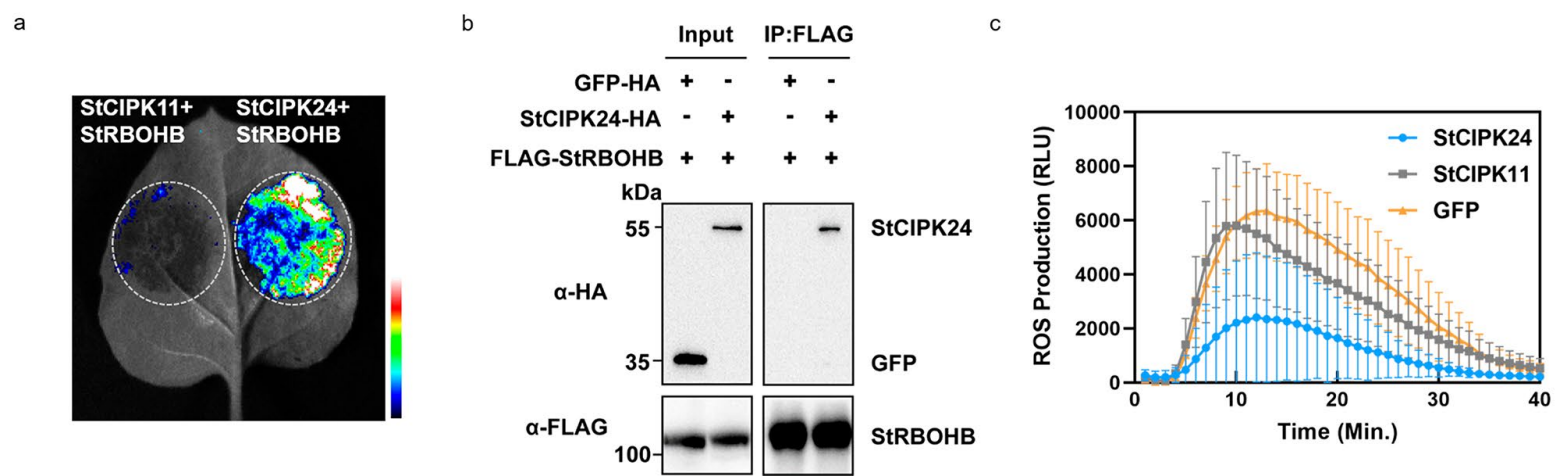
StCIPK24 associates with StRBOHB and negatively regulate flg22-induced ROS production. (**a**) StCIPK24 associates with StRBOHB in the split-luciferase assay. Constructs carrying StCIPK24-Cluc and Nluc-StRBOHB were co-expressed in *N. benthamiana* leaves for 2 days. The infiltrated leaf was detached and treated with 1 mM luciferin, and the bioluminescence image was captured by a CCD camera. The StCIPK11 was used as a negative control. The pseudo-color bar shows the range of luminescence, indicating the interaction intensity. (**b**) The association between StCIPK24 and StRBOHB in *N. benthamiana* were detected by co-immunoprecipitation. StCIPK24-HA or GFP-HA was co-expressed with FLAG-StRBOHB in *N. benthamiana* for 36 h. Total proteins were immunoprecipitated with an α-FLAG antibody (α-FLAG IP) and immunoblotted with α-HA (IB: α-HA) or a-FLAG (IB: α-FLAG; top two panels). Protein inputs are shown with immunoblotting before immunoprecipitation (bottom two panels). (**c**) Overexpression of *StCIPK24*, but not *StCIPK11*, in *N. benthamiana* suppressed flg22-induced ROS production. The indicated *StCIPKs* were transient expressed in *N. benthamiana* for 2 days, and subjected to flg22-induced ROS examination. The infiltrated leaf discs were treated with 1µM flg22 and the ROS production was measured as relative light units (RLU) by a luminometer. The data are shown as mean ± SD (n ≥ 12 leaf discs)

The above results indicated that StCIPK7 negatively regulates the resistance to *P. infestans*. Nevertheless, it did not play a role in the PTI and ETI as StCBL3 did. Multiple lines of evidence have showed that the apoplastic ROS accumulation is mainly mediated by RBOHs protein regulated by diverse kinases [24]. Additionally, our aforementioned results have shown that StCBL3 negatively regulate ROS production. Therefore, we speculate that StCBL3 may act together with another StCIPK to modulate ROS production. To identify the potential StCIPK involving in the regulation ROS production, we first test the association between StCIPKs and StRBOHB. Interestingly, we found that StCBL3-interacting StCIPK24, not other StCIPKs, exhibits strong association with StRBOHB in the split-luciferase complementation assays (Fig. 7a and S6). The coIP assay in *N. benthamiana* further confirmed that StCIPK24 associates with StRBOHB (Fig. 7b). Moreover, transient overexpression of *StCIPK24* inhibited flg22-induced ROS production compared with the *GFP* control and *StCIPK11* (Fig. 7c). Together, the results suggest that that StCBL3-StCIPK24 negatively regulate ROS production by interacting with StRBOHB protein.

## Discussion

*P. infestans* is an oomycete pathogen that naturally infects the crop plants potato and tomato, and has been wildly used in the interaction with the model plant *N. benthamiana* in the laboratory [31–33]. It has been shown that combinations of CBLs and CIPKs function as a signaling node in response to environmental stimuli [34–38]. However, the biological roles of CBL-CIPK have not been explored in potato-*P. infestans* system. In this study, via the transcriptome analysis of the *P. infestans*-infected potato plants combined with fast-forward genetic VIGS screen, we systemically screen the potato CBLs involved in plant immunity and identified StCBL3 as a novel negative regulator playing a vital role in regulating multiple layers of plant defense response to *Phytophthora* infection. StCBL3 negatively regulates multiple PAMPs-induced responses, such as flg22-induced ROS burst and XEG1-induced cell death (Fig. 2a and b, S2a and S2b). Interestingly, our results showed that overexpression of *StCBL3* did not affect the cell death triggered by INF1, but inhibited flg22-incued ROS burst. This observation indicate that the underlying mechanism of INF1-triggered immune responses are partially different with that induced by flg22. To support this hypothesis, it has been reported that MAPK-WRKY pathway is involved in INF1-indued ROS burst, but not in the flg22-ROS burst [39]. Additionally, systemic investigation of the roles of soybean malectin/malectin-like domain-containing receptor-like kinases (MRLKs) in the plant immunity revealed that GmMRLKs differentially modulate PTI and ETI. For example, GmMRLK20 and GmMRLK25 specifically regulate INF1-induced cell death, but not flg22-induced ROS production [40]. However, the exact mechanism for the same component(s) differently responding to diverse PAMPs is still obscure. It will be interesting to investigate the functional specificity of StCBL3 involved in flg22- but not INF1-mediated immune singling in the future. StCBL3 also plays a negative role in regulating Avr3a/R3a-mediated cell death (Fig. 2c and d). Therefore, we propose that StCBL3 act in a convergent regulatory node in both PTI and ETI, which further potentiates the crosstalk between PTI and ETI.

The transcriptome analysis and our RT-qPCR data on the StCBL3 and StCIPK7 verify that the expression of *StCBL3* and *StCIPK7* are induced upon *P. infestans* infection. Previous studies have shown that the up-regulation of host genes during infection may indicate the exploitation of cellular resources and/or the activation of defense responses [41]. However, our multi-dimensional phenotypic analysis demonstrate that StCBL3-StCIPK7 acts as a negative module in plant immunity. Several studies have shown that CBL-CIPK appears to be positive components in plant immunity. For instance, tomato SlCBL10-SlCIPK6 module are positive regulator in multiple effectors-NLRs-induced cell death [20]. Wheat TaCBL4-TaCIPK8 positively regulate *Pst*resistance [21]. The negative role of StCBL3-StCIPK7 in plant immunity indicates that the StCBL3-StCIPK7 module may evolved for benefit of *P. infestans* infection. It is possible that StCBL3 is hijacked by the unknown effector secreted by *P. infestans* to promote the infection. This hypothesis is still needed to explore more clues.

Our studies reveal a functional role for CBL-CIPK module in the regulation to disease resistance to *P. infestans.* Salicylic acid (SA), a key signaling molecule of plant resistance against biotrophs, induces the expression of *PR* genes and results in systemic acquired resistance (SAR). Transcript level of *PR1*, marker gene of the SA signaling pathway was reduced in *StCBL3* transgenic plants (Fig. 3g). StCBL3-StCIPK7 module probably work in plant immunity by governing the defense genes expression. In agreement with our hypothesis, several studies have indicated that different Ca^2+^ sensors modulate the elevation in SA that occurs late in immune responses [42]. In *Arabidopsis*, SA accumulation and *PR* gene expression are regulated by CPK1 [43]; The CAM-binding transcription activator AtCAMTA3 (also known as AtSR1) regulates SA-mediated plant immunity [44]. Wheat TaCIPK10 interacts and phosphorylates NH2, homologous to *Arabidopsis* NPR3/4 and master regulator in SA signaling pathway, to regulate *Pst* resistance [22]. Following treatment of rice cells with the fungal MAMP Trichoderma viride/ethylene-inducing xylanase (TvX/EIX), OsCIPK14/15 functions to promote *PR* gene expression, phytoalexin biosynthesis, and cell death, probably by combination with the Ca^2+^ sensor OsCBL4 [45]. Thus, we infer that SA-mediated plant immunity in potato is negatively modulated by an activated StCBL3-StCIPK7 complex. Further investigation of this important signaling node will shed light on the immunity activation following *P. infestans* infection.

Unlike StCBL3, StCIPK7 plays a specific role in regulating *Phytophthora* resistance, but not in other immune responses, such as PAMP-induced ROS production and cell death, and Avr3a/R3a-induced cell death. StCIPK7 might interact and phosphorylates the unknown immune-related transcriptional factors. It will be interesting to dissect the transcriptional reprogramming regulate by StCIPK7 in the future. Although we performed the systemic screening by transient expression CIPKs on *N. benthamiana*. It can not rule out the possibility that some StCIPKs, which might be overlooked in this study as result of functional redundancy, play roles in PTI and ETI. It will be interesting to further explore the roles of other StCIPKs in plant immunity.

CBLs bind to CIPKs to activate CIPKs that in turn phosphorylate CBLs to enhance the functionality of CBLs. Consistently, our biochemical characterization for StCBL3 and StCIPK7 indicated that StCIPK7 is an active kinase, whose activity is positively regulated by StCBL3 and Ca^2+^. Several studies have shown that the C terminal region of CBLs can be directly phosphorylated by CIPKs at some conserved serine/threonine residues [46–49]. This phosphorylation is thought to be functionally important for the specificity and activity of CBL-CIPK complexes to regulate their downstream targets.

The negative role of StCBL-StCIPK module makes it as susceptible (S) gene module. The usage of *S* gene has been emerged as an important mean to engineer the crop with broad and durable resistance to different pathogens, as the genome editing technology has become powerful tool because of its high efficiency, relatively low cost, and ease of use [50]. For example, introducing a novel allele of wheat *S* gene *MLO* into elite wheat varieties by precision genome editing enable confer durable resistance to powdery mildew [51]. Additionally, CRISPR/Cas9-mediated gene editing in wheat kinase gene *TaPsIPK1* (another *S* gene) confers robust rust resistance without growth and yield penalty [52]. Our work highlights a novel CBL-CIPK module regulating potato immune response and provide a valuable gene resource to engineer potato resistance to *Phytophthora* pathogens. Therefore, it will be worthwhile to precise editing the StCBL-StCIPK module in potato to fulfil the green prevention and control in the future.

Besides StCBL3-StCIPK7 module negatively regulates the disease response to *Phytophthora* pathogens, our results showed that ROS generation is regulated by StCIPK24 which interacts and probably phosphorylates StRBOHB protein. Furthermore, our data showed that StCBL3 also interacts with StCIPK24 in yeast. Therefore, we speculate that StCBL3-StCIPK24-StRBOHB cascade mediates the ROS burst. It has been documented that the large membership of the CBL and CIPK gene families in flowering plants constitute a highly convoluted and sophisticated signaling network [17]. Each CBL interacts with a subset of CIPKs and each CIPK interacts with one or more CBLs [53]. Such specificity and overlap in CBL-CIPK interactions result in their signaling specificity and functional synergism. However, the precise mechanism of StCIPK24 regulating StRBOHB protein and ROS production remains to be investigated in the future. In agree with our result, the wheat TaCIPK5 interacts with TaRBOHi to positively regulate ROS production [21]. In tomato, ROS production is regulated by CBL10/CIPK6 [20]. Taken together, our study supports a role for CBL-CIPK in contributing to ROS production, adding another level to fine-tune ROS level in response to pathogen attacks.

## Conclusions

Based on our results, we propose a model that pathogen-secreted effector may hijack StCBL3, which interacts with StCIPK7, thereby enhancing its kinase activity. StCIPK7 phosphorylates the unknown substrate, possible transactional factor, depressing the defense genes expression contributing to the resistance response. On the other hand, StCBL3 couples with StCIPK24 to regulate PAMP-induced ROS burst, probably by phosphorylating StRBOHB protein and negatively regulate its activity. Overall, the data that we present serve as a basis towards advancing our understanding of the role of the CBL-CIPK complex during oomycete-pathogen infection.

## Materials and methods

### Plant materials, growth condition, *Phytophthora* culture and infection assays

*N. benthamiana* plants were grown and maintained in a growth chamber at 25 °C under 16 h light/8 h dark photoperiod with a 60% relative humidity.

The *P. infestans* strain MZ (isolated from Heilongjiang province, China) was cultured on Rye A medium (1 L distilled water containing 60 g rye extract, 20 g sucrose and 15 g agar) at 18 °C in the dark for 10–12 days. *P. capsici* isolate LT263 used was routinely cultured at 25 °C on 10% vegetable (V8) juice medium in the dark.

### *Phytophthora* infection assays

For *P. infestans* inoculations, the sporangia suspension solution was made in 50 mL centrifuge tube by mixing 5 mL sterile pre-cooled water with the mycelium and quantified using a hemocytometer, and the concentration was adjusted to 350 sporangial/10 µL with pre-cooled water. The fully expanded *N. benthamiana* leaves were inoculated with 10 µL droplets of a freshly prepared sporangia suspension and then were placed under controlled environmental conditions (18 °C darkness for 1 day, then 18 °C 16 h of light and 8 h of darkness for 6 days) before phenotypes were scored.

For *P. capsici* inoculations, the mycelial plugs were incubated in liquid V8 medium for 3 days, washed three times with sterilized water, and incubated in water until sporangia formed. To release the zoospores, the cultures were incubated at 4 °C for 30 min, followed by incubation at 25 °C for 1 h. The zoospore concentration was determined using a haemocytometer. Detached *N. benthamiana* leaves were inoculated with zoospores at the number of 350. Inoculated leaves were photographed under UV light 36 h post inoculation, and the lesion areas were measured using ImageJ [54].

### Plasmid construction and generation of transgenic plants

Constructs for transient expression in *N. benthamiana* were generated by amplifying corresponding sequences and inserting into the pCAMBIA-1300-35 S vectors with different tags, including 3xFLAG, HA, NLUC and CLUC. The HA tag was incorporated into NLUC vector. All the constructs with a single insertion were generated by ClonExpress II one-step cloning kit (Vazyme Biotech, Nanjing, China) and verified by Sanger sequencing.

*Agrobacterium*-mediated transformation of *N. benthamiana* was carried out to obtain stable transgenic lines. Multiple transgenic lines for *StCBL3* overexpression were analyzed by immunoblotting for protein expression. T3 generation of *N. benthamiana* were selected to obtain homozygous seeds for further studies.

The primers used to generate these constructs are listed in Supplemental Table 1.

### Identification and sequence analysis of *CBL* gene family in potato

The TAIR v10 (http://www.arabidopsis.org/) and DM v6.1 (http://spuddb.uga.edu/) databases were used to collect amino acid sequences of CBLs in Arabidopsis and potato (Solanum tuberosum L.). To identify all StCBLs, the 10 *Arabidopsis* CBLs protein sequences were applied as queries by BLAST (http://spuddb.uga.edu/blast.shtml), with E-value < e^− 5^. Then the typical CBL domains EF-hand domains (PF13499 or PF13833) were queried via an HMM search. Each StCBL protein was also examined by SMART (http://smart.embl-heidelberg.de/). All protein sequences identified by these methods were complied, eliminated the redundant sequences. The candidate StCBLs were analyzed the MW and pI in ExPASy (https://web.expasy.or-g/prot-param/).

Phylogenetic trees were generated based on the alignment results using the maximum likelihood method and 1,000 bootstrap trials with the MEGA 7.0 software. For the heatmap, normalized gene expression values FPKM (fragments per kilobase of exon per million fragments mapped) were first transformed using log_2_ (FPKM + 1) and then plotted by using pheatmap package in R.

### Virus-induced gene silencing (VIGS) assay

Plasmids containing binary TRV vectors pTRV-RNA1 and pTRV-RNA2 derivatives were introduced into Agrobacterium tumefaciens strain GV3101 by electroporation. Bacterial cultures were prepared as described previously [55]. Cells were pelleted by 4200 rpm centrifugation, re-suspended in a solution containing 10 mM MgCl_2_, 10 mM MES and 200 µM acetosyringone, adjusted to OD600 = 0.3 and incubated at 25 °C for at least 3 h. Bacterial cultures containing pTRV-RNA1 and pTRV-RNA2 derivatives were mixed at a 1:1 ratio and inoculated into the true leaves of two-week-old soil-grown plants using a needleless syringe.

### RNA isolation, reverse transcription and RT-qPCR analysis

Total RNA was extracted by Trizol reagent (Zomanbio) and quantified with a spectrophotometer (NanoDrop2000, Thermo Fisher Scientific). 1 µg of total RNAs were reverse-transcribed to synthesize the first-strand cDNA with Reverse Transcription Kit (TaKaRa). RT-qPCR was carried out using 2×RealStar SYBR qPCR Mix (GeneStar) on ABI QuantStudio 6 Flex Real-time PCR System (Applied Thermo Fisher) with gene-specific primers (Supplementary Table 1) following a manufactural protocol. The relative levels of gene expression were calculated using the 2^(−△△Ct)^ method with *StActin* or *NbActin* used as an internal reference gene.

### Cell death examination and electrolyte leakage assay

To analyze the effect of StCBL3 and StCIPK7 on multiple PAMPs- and Avr3a/R3a-induced cell death, *Agrobacterium* which carried *StCBL3* or *StCIPK7* was first infiltrated into *N. benthamiana* leaves. *Agrobacterium* carried the pCAMBIA1300-GFP-HA empty vector as a control. The same areas were infiltrated with *Agrobacterium* with XEG1, INF1 or Avr3a/R3a 24 h later. For the cell death analysis in stable StCBL3 transgenic plants, the Agrobacterium carrying Avr3a/R3a was directly infiltrated into the leaves. The cell death phenotype was visualized and photographed under UV light 2–4 days later.

To examine the cell death by electrolyte leakage assay, leaf disks (1 cm diameter) were taken and floated with 5mL distilled water, incubated at room temperature for 3 h. The ion leakage was measured with conductivity meter (METTLER TOLEDO, Switzerland) as “value A”. Then the samples were boiled for 30 min, cooled to room temperature, the conductivity was measured again to get “value B”. For each sample ion leakage was exhibited as (value A / value B) *100%.

### Split-luciferase complementation assay in *N. benthamiana*

The split-luciferase complementation assay was based on previous report [56]. Cluc- and Nluc-HA tagged vectors were transformed in *Agrobaterium*, and then transiently co-expressed in *N. benthamiana* for 36 h. The infiltrated leaves were sprayed with 1 mM luciferin buffer for 5 min and visualized with a Tanon-5200 Multi Chemiluminescent Imaging System (Tanon Science & Technology Co., Ltd). The luminescence intensity was measured using microplate luminometer Tecan F200.

### Yeast two-hybrid assays

Yeast two-hybrid assays were performed with the Matchmaker Gold yeast two-hybrid system. StCIPKs and StCBLs were cloned into pGBKT7 and pGADT7 vectors, respectively. Then pGBKT7-StCIPKs and pGADT7-StCBLs were co-transformed into Golden yeast strain using the lithium acetate method. Transformants were first selected on double synthetic dropout medium lacking tryptophan and leucine (TaKaRa), then selected on quadruple dropout medium lacking tryptophan, leucine, histidine and adenine (TaKaRa) to detect protein interactions. pGBKT7 and pGADT7 empty vector were used as the negative control.

### Co-immunoprecipitation (Co-IP) assay

The HA- and FLAG-tagged constructs were transiently expressed in *N. benthamiana* leaves by agroinfiltration for 48 h. The infiltrated leaves were harvested and total protein was extracted with lysis buffer (150 mM NaCl, 1 mM EDTA, 25 mM Tris-HCl pH7.5, 10% glycerol, 2% PVPP, 10 mM DTT, 1×protease inhibitor, 0.5% Triton). After vortexed vigorously for 30 s, the sample was placed on ice for 10 min and then centrifuged at 13,000 g, 4 °C for 15 min, and the supernatant was incubated with agarose-conjugated anti-FLAG beads (Sigma) for 2 h with gentle shaking, The beads were collected and washed four times with washing buffer (150mM NaCl, 1mM EDTA, 25mM Tris-HCl pH7.5, 10% glycerol, 5mM DTT). The beads were boiled with SDS-PAGE loading buffer at 94 °C for 10 min to elute the proteins. Immunoprecipitated and input proteins were analyzed by immunoblots with anti-HA and anti-FLAG indicated antibodies, respectively.

### In Vitro pull-down and phosphorylation assays

The His- and GST-fused recombinant proteins were expressed in *E. coli* strain BL21 (DE3) and purified using Ni-NTA agarose (LABLEAD), glutathione agarose (LABLEAD), respectively, according to each manufacturer’s instructions. Approximately 10 µg of GST or GST-fused proteins was incubated with 5 µL of prewashed glutathione agarose beads in 1 mL of pull-down buffer (20mM Tris-HCl pH7.5, 150 mM NaCl, 0.1 mM EDTA, and 0.5% Triton-X100) for 30 min at 4 °C with gentle shaking. The beads were harvested by centrifugation at 1000 g for 1 min and inoculated with 10 µg of His-fused proteins in 1 mL of pull-down buffer for 1 h at 4 °C with gentle shaking. Finally, the beads were harvested and washed three times with 1mL of washing buffer (20 mM Tris-HCl pH 7.5, 300 mM NaCl, 0.1 mM EDTA, and 0.1% Triton X-100). The pulled-down proteins were released from beads by boiling in SDS-PAGE loading buffer and analyzed by immunoblotting with anti-HIS and anti-GST antibodies (TransGen), respectively.

The in vitro phosphorylation reactions were performed at 25 °C for 2 h in kinase buffer (12.5 mM Tris-HCl pH 7.5, 5 mM MgCl_2_, 0.5 mM CaCl_2_, and 0.5 mM DTT) containing 10 µg of substrate proteins, 1 µg of kinases, and 0.1 mM ATP with or without 1 µCi of [^32^P]-γ-ATP. Negative control reactions with the addition of 50mM EGTA were included to confirm the specificity of StCIPK7-mediated phosphorylation. The reactions were stopped by adding SDS loading buffer, and the phosphorylation of recombinant proteins was analyzed by autoradiography (Typhoon 9410 imager) after protein separation by SDS-PAGE.

### Reactive oxygen species burst assay

The indicated constructs were transiently expressed in *N. benthamiana* leaves by agroinfiltration for 48 h. The leaf disks were collected and incubated overnight with 200 µL ultra-pure distilled water in a 96-well white plate to eliminate the wounding effect. Water was replaced by 100 µL of a reaction solution containing 100 µg/mL luminol (Sigma) and 1 mg/mL horseradish peroxidase (Sigma) supplemented with 1µM flg22 (Sangon). Luminescence was measured with a microplate luminometer (TECAN, Männedorf, Switzerland) for a period of 40 min. ROS production was indicated as means of relative light units (RLU).

### Electronic supplementary material

Below is the link to the electronic supplementary material.

Supplementary Material 1

Supplementary Material 2

## Data Availability

The datasets used and/or analyzed during the current study, are available from the corresponding author on reasonable request.

## Abbreviations

CBLs: Calcineurin B-Like proteins
CIPKs: CBL-interacting protein kinases
ROS: Reactive oxygen species
VIGS: Virus-induced gene silencing
PTI: Pattern-triggered immunity
ETI: Effector-triggered immunity
NLR: Nucleotide-binding domain leucine-rich repeat
HR: Hypersensitive response
CDPKs/CPKs: Calcium dependent protein kinases
*Pst*: *Puccinia striiformis* f.sp. *tritici*
RLU: Relative light units
