# Supplementary material for: Potato calcium sensor modules StCBL3-StCIPK7 and StCBL3-StCIPK24 negatively regulate plant immunity
Source: BMC Plant Biol. 2024 Jan 5;24:30. doi: 10.1186/s12870-023-04713-x (PMC10768403; doi:10.1186/s12870-023-04713-x)

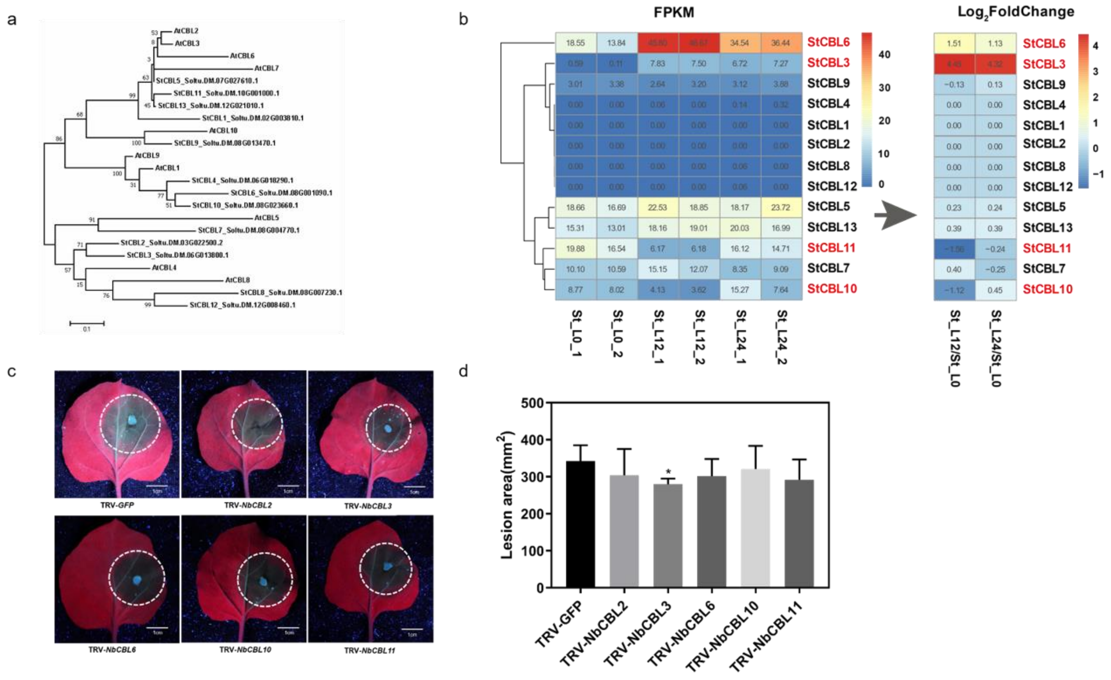

Fig. S1. Phylogenetic and transcriptome analysis for StCBLs, and silencing *NbCBL3* in *N. benthamiana* reduced the lesion size caused by *P. capsici* infection.

(a) Phylogenetic relationship of CBLs from *Arabidopsis* and potato. The maximum likelihood tree was constructed using MEGA 7.0. The number above the internal branches indicates bootstrap values estimated based on 1,000 bootstrap replications. Branches are labeled with protein names and potato gene ID. The scale bar represents 10% weighted sequence divergence.

(b) The heatmap graph showed all the *StCBLs* expression in response to *P. infestans* infection at 0 h, 12 h and 24 h with two biological replicates. Red color indicates relatively high expression and blue indicates relatively low expression. Left panel: the corresponding value of fragments per kilobase of transcript per million is shown in the colored box. Right panel: the log_2fold change value calculated from left panel is shown. The differential expressed *StCBLs* genes are labeled in red color.

(c and d) NbCBL3 was identified to negatively regulate disease resistance to *P. capsici*

infection via VIGS system-based inoculation assays. Representative images of *P. capsici* lesions on TRV-*GFP* and indicated TRV-*NbCBLs* silenced leaves. Leaf images were taken under UV light at 36 h post inoculation, scale bar = 1cm (c). Meanwhile, lesion area was quantified by ImageJ (d). The data are shown as mean ± SD ($n \geq 6$ leaves from different plants of each genotype), and the asterisk indicates a significant difference with a Student's *t*-test (*$P < 0.05$). The experiments were independently repeated 3 times with similar results.

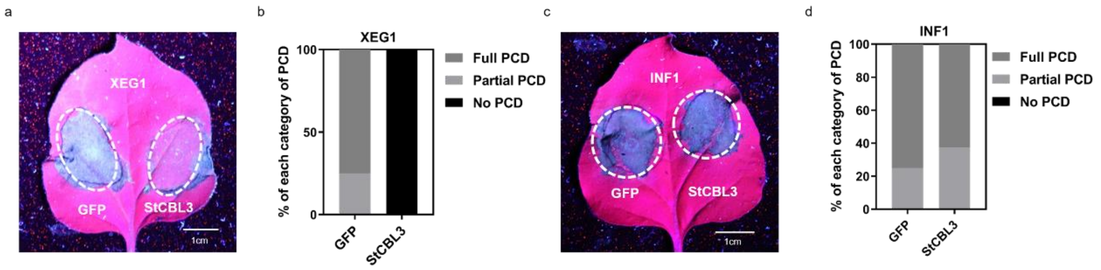

Fig. S2. Overexpression of *StCBL3* in *N. benthamiana* suppresses XEG1-induced cell death, but not INF1-induced cell death.

(a and b) XEG1-induced cell death was dramatically inhibited by the expression of *StCBL3.* Cell death was visualized and photographed under UV light 48 h after infiltration, scale bar = 1 cm (a). The cell death extent was classified according to its extent: full PCD; partial PCD; no PCD (b). The experiments were independently repeated 3 times (each time with 6 biological replicates) with similar results.

(c and d) INF1-induced cell death in *StCBL3*-overexpressed plants was similar with that in *GFP*-overexpressed plants. Cell death was visualized and photographed under UV light 48 h after infiltration, scale bar = 1 cm (c). The cell death extent was classified according to its extent: full PCD; partial PCD; no PCD (d). The experiments were independently repeated 3 times (each time with 6 biological replicates) with similar results.

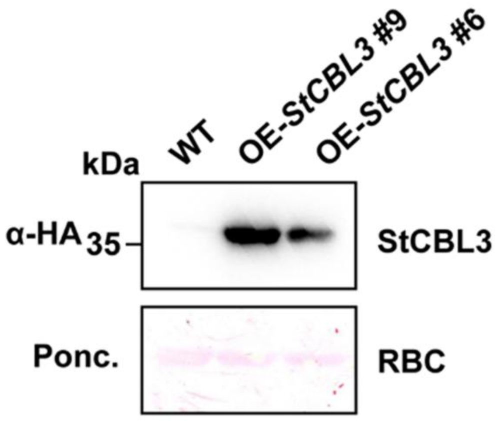

Fig. S3 Two transgenic lines of *StCBL3* used for phenotypic assay were analyzed by immunoblotting for protein expression. Top panel: the protein expression in *N. benthamiana*. Bottom panel: protein loading is indicated by Ponceau stain (Ponceau S).

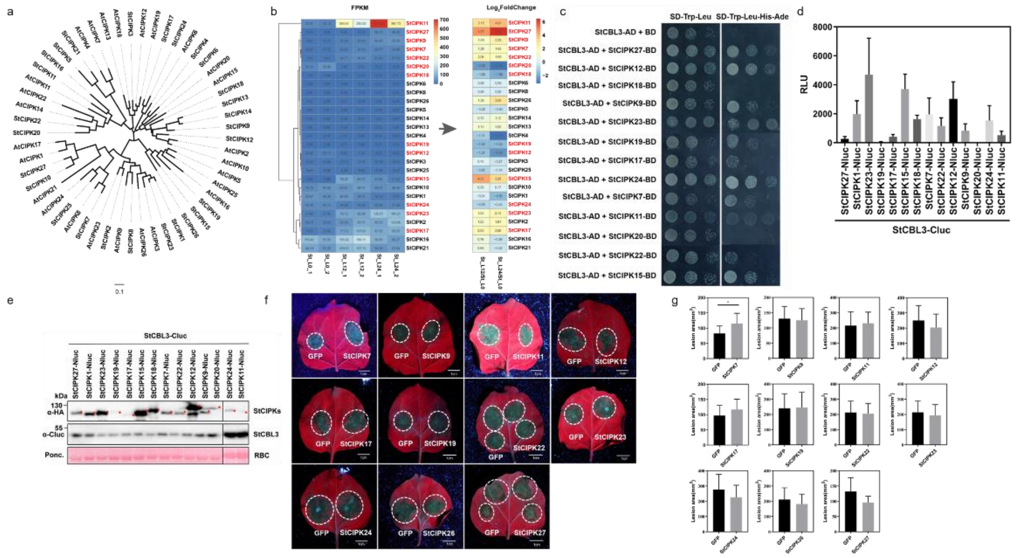

Fig. S4. StCIPK7 was identified to be involved in defense to *P. infestans* infection via diverse genetic and biochemical approaches

(a) Phylogenetic relationship of CIPKs from *Arabidopsis* and potato. The maximum likelihood tree was constructed using MEGA 7.0. Branches are labeled with protein names. Bootstrap percentage support for each branch is indicated. The scale bar represents 10% weighted sequence divergence.

(b) The heatmap graph showed all the *StCIPKs* expression in response to *P. infestans* infection at 0 h, 12 h and 24 h with two biological replicates. Red color indicates relatively high expression and blue indicates relatively low expression. Left panel: the corresponding value of fragments per kilobase of transcript per million is shown in the colored box. Right panel: the log_2fold change value calculated from left panel is shown. The differential expressed *StCIPKs* genes are labeled in red color.

(c) StCBL3 interacts with multiple StCIPKs in yeast. The interaction between pAD-StCBL3 and pBD-empty vector or indicated pBD-StCIPKs was tested on tested on synthetic defined medium without leucine, tryptophan, histidine and adenine (SD-L-T-H-A). Serial dilutions of the yeast colonies were plated.

(d and e) StCBL3 associates with multiple StCIPKs in split-luciferase assay. Constructs carrying StCBL3-Cluc and StCIPK7-Nluc were co-expressed in *N. benthamiana* leaves for 2 days. The infiltrated leaf discs were detached and treated with 1 mM luciferin. The protein interaction intensity (right panel) is shown by the relative luminescence unit (RLU) (Mean ± SD, $n \geq 12$, n represents sample number) (d). The StCBL3 and StCIPKs were immunoblotted with anti-Cluc and anti-HA antibody, respectively. Protein loading is indicated by Ponceau stain (Ponceau S) (e).

(f and g) Transient expression of *StCIPK7* in *N. benthamiana* leaves enhanced *P. infestans* colonization compared to half leaves inoculated with *Agrobacterium* expressing *GFP* empty vector. Transient expression of 11 CIPKs followed by infection assay were performed. Leaf images were taken at 6 days post inoculation, scale bar = 1cm (f). Lesion area was quantified by ImageJ (g). The data are shown as mean ± SD ($n \geq 6$ leaves from different plants), and the

asterisk indicates a significant difference with a Student's *t*-test (*$P < 0.05$). The experiments were independently repeated 3 times with similar results.

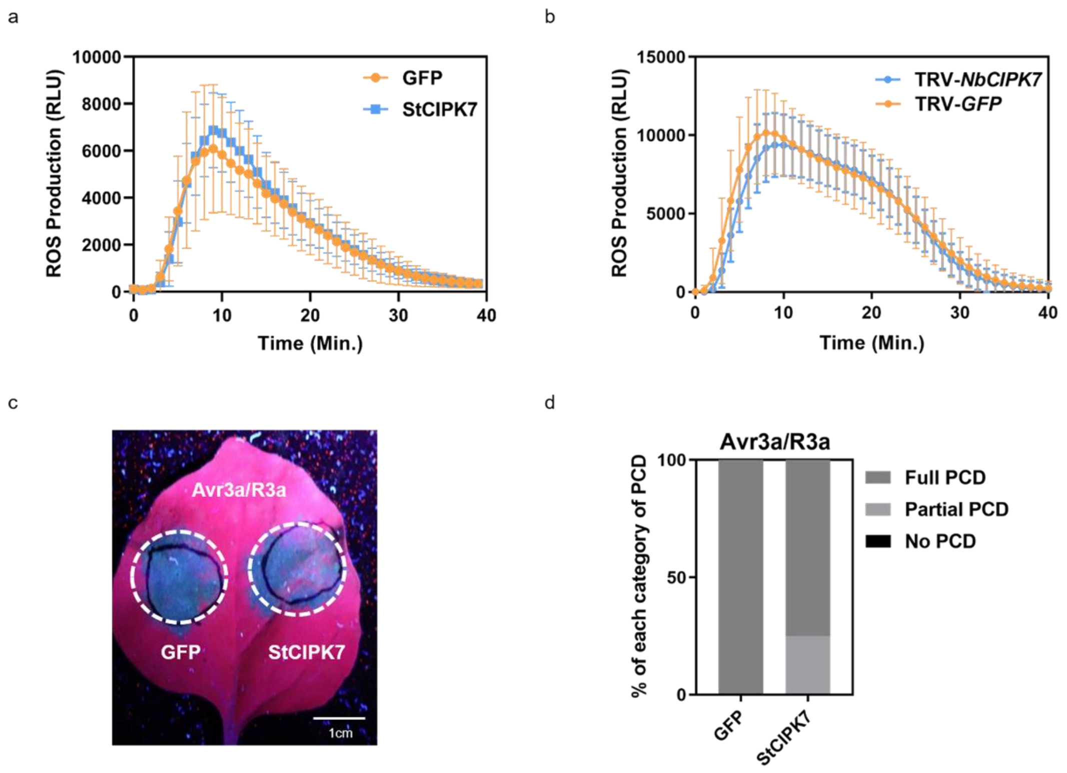

Fig. S5. StCIPK7 does not have effect on flg22-induced ROS and Avr3a/R3a-induced cell death

(a) No difference was observed in ROS burst induced by flg22 in *NbCIPK7*-silenced plants compared with the control TRV-*GFP* plants. Leaf discs were treated with 1μM flg22 and ROS production was measured as relative light units (RLU) by a luminometer. The data are shown as mean ± SD (n ≥ 12 leaf discs).

(b) The flg22-induced ROS burst in *StCIPK7*-overexpressed plants is similar with that in *GFP*-overexpressed plants. Leaf discs were treated with 1μM flg22 and ROS production was measured as relative light units (RLU) by a luminometer. The data are shown as mean ± SD (n ≥ 12 leaf discs).

(c and d) Avr3a/R3a-induced cell death in *StCIPK7*-overexpressed plants is similar with that in *GFP*-overexpressed plants. Cell death was visualized and

photographed under UV light 48 h after infiltration, scale bar = 1 cm (c). The cell death extent was classified according to its extent: full PCD; partial PCD; no PCD (d). The experiments were independently repeated 3 times (each time with 6 biological replicates) with similar results.

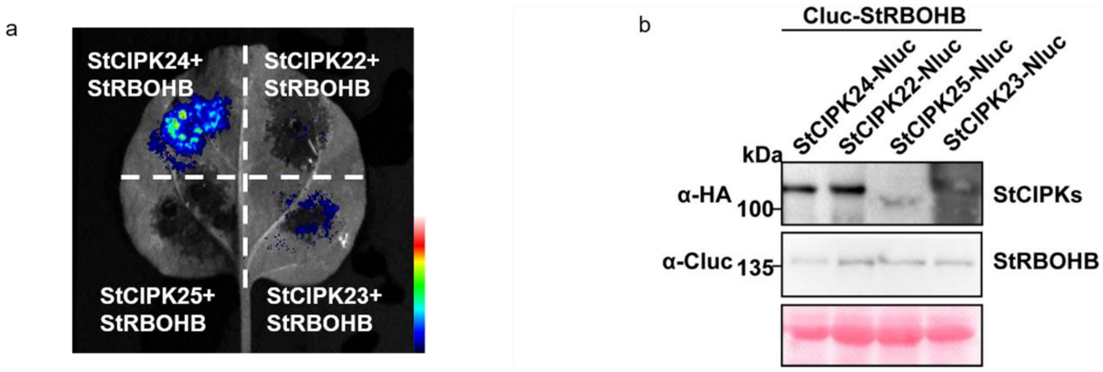

Fig. S6. StCIPK24 associates with StRBOHB in the split-luciferase assay.

(a) Constructs carrying StCIPK24-Cluc, StCIPK22-Cluc, StCIPK25-Cluc, StCIPK23-Cluc and Nluc-StRBOHB were co-expressed in *N. benthamiana* leaves for 2 days, respectively. The infiltrated leaf was detached and treated with 1 mM luciferin, and the bioluminescence image was captured by a CCD camera. The pseudo-color bar shows the range of luminescence, indicating the interaction intensity.

(b) The indicated StCIPKs and StRBOHB were immunoblotted with anti-HA and anti-Cluc antibody, respectively. Protein loading is indicated by Ponceau stain (Ponceau S).

Uncropped images for gels

Figure 1f

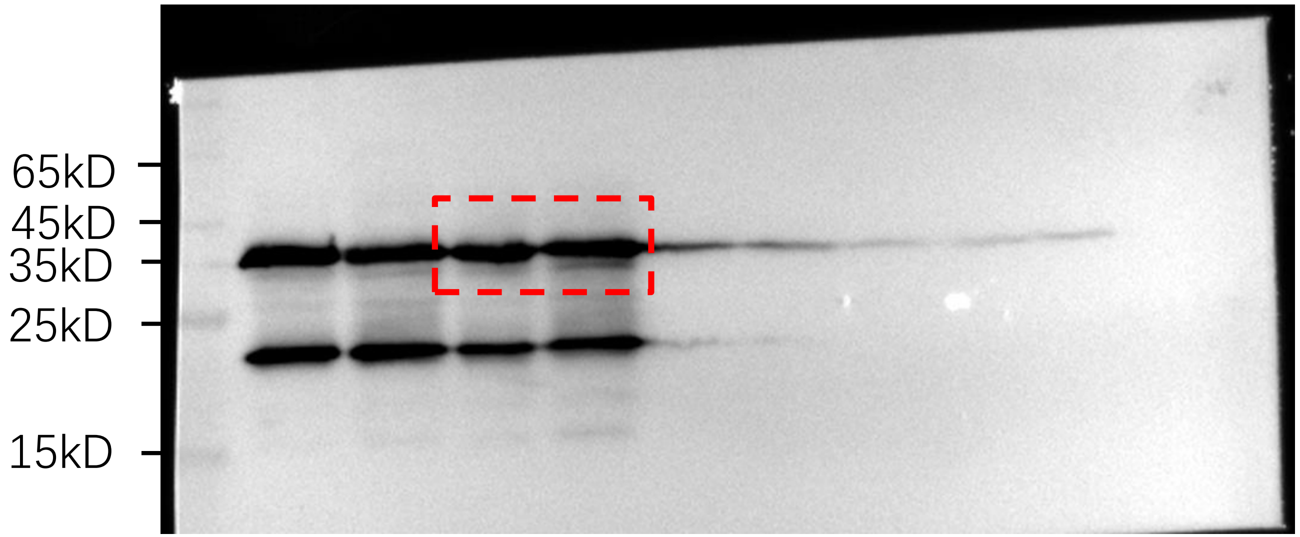

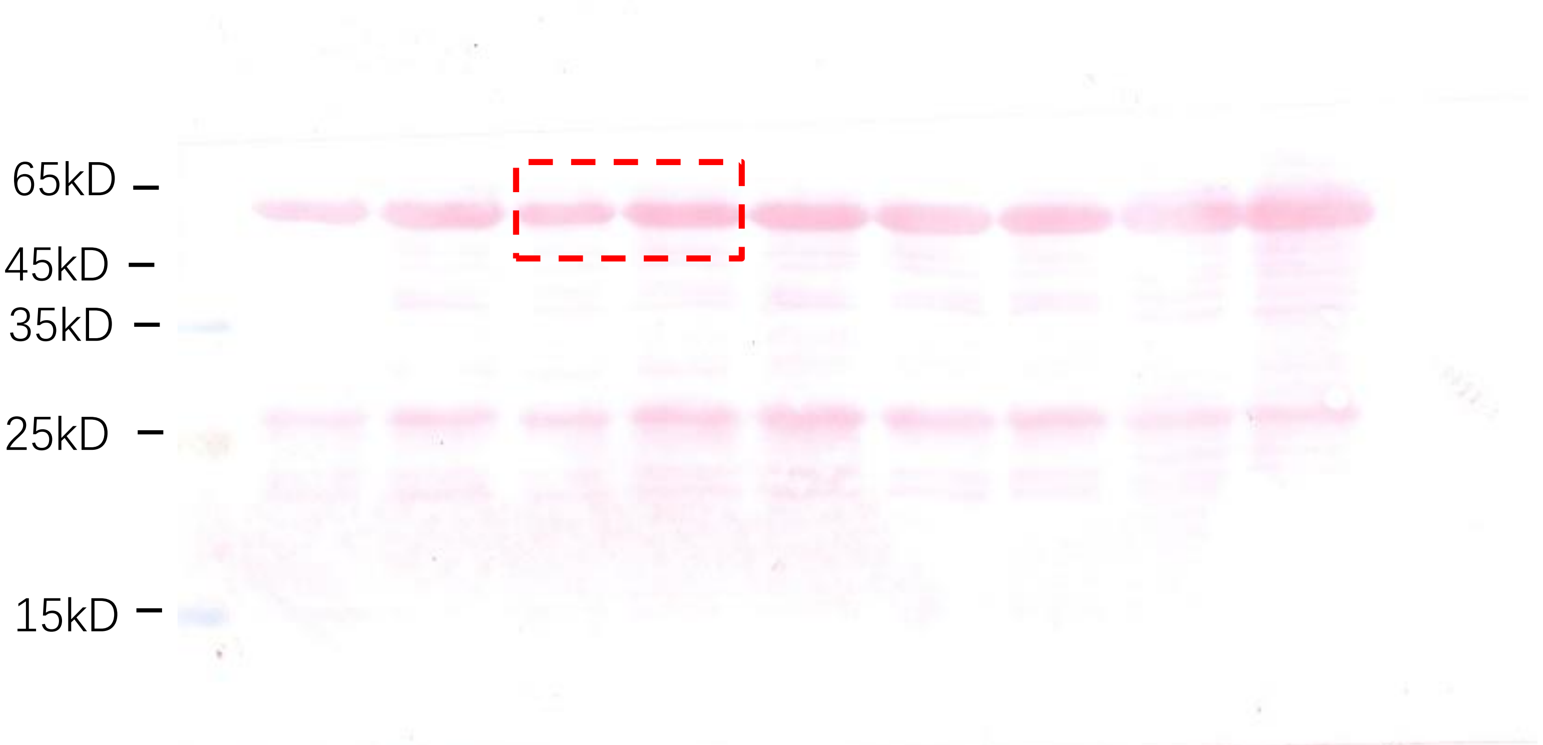

Uncropped images for gels

Figure 5c

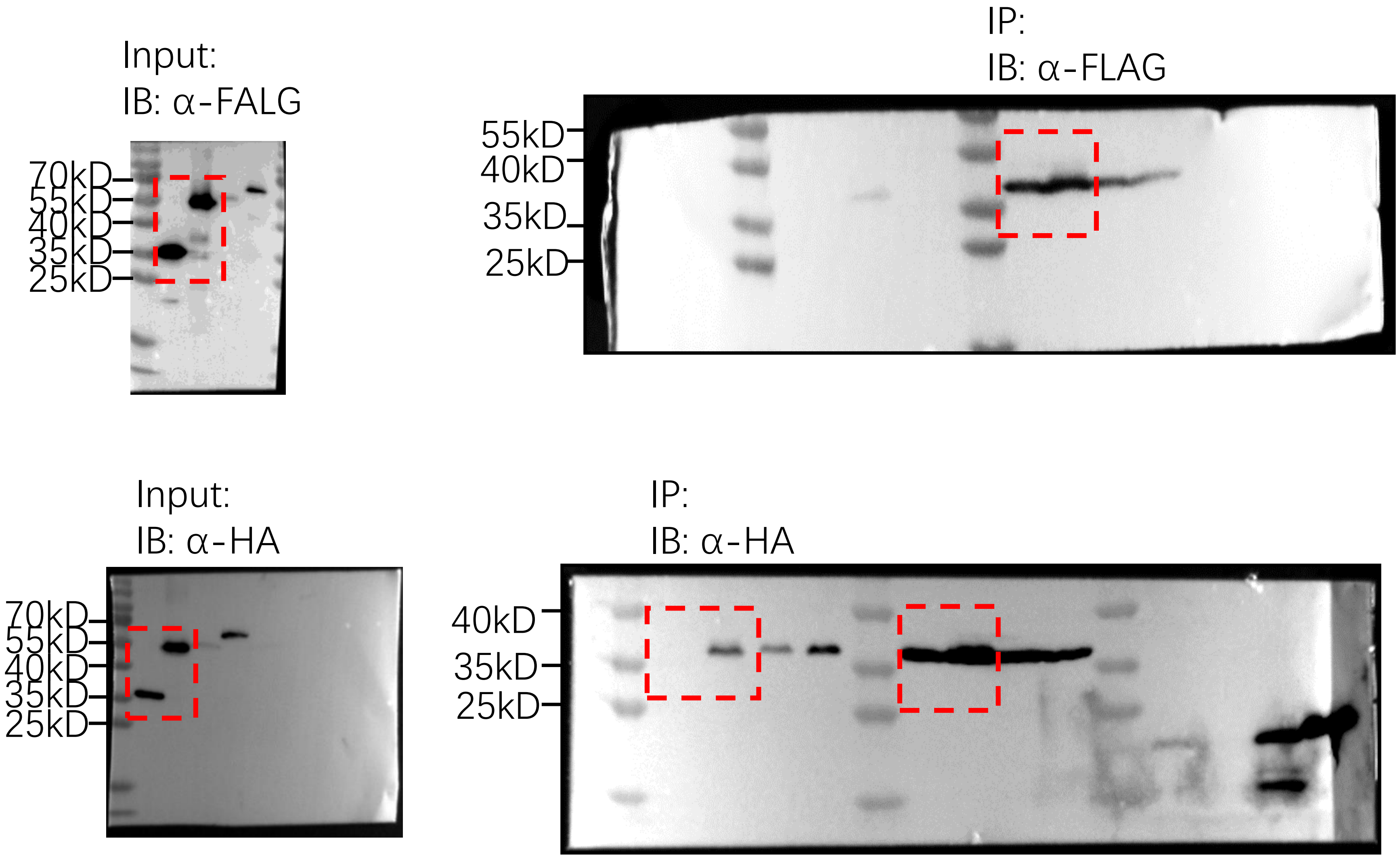

Uncropped images for gels

Figure 5d

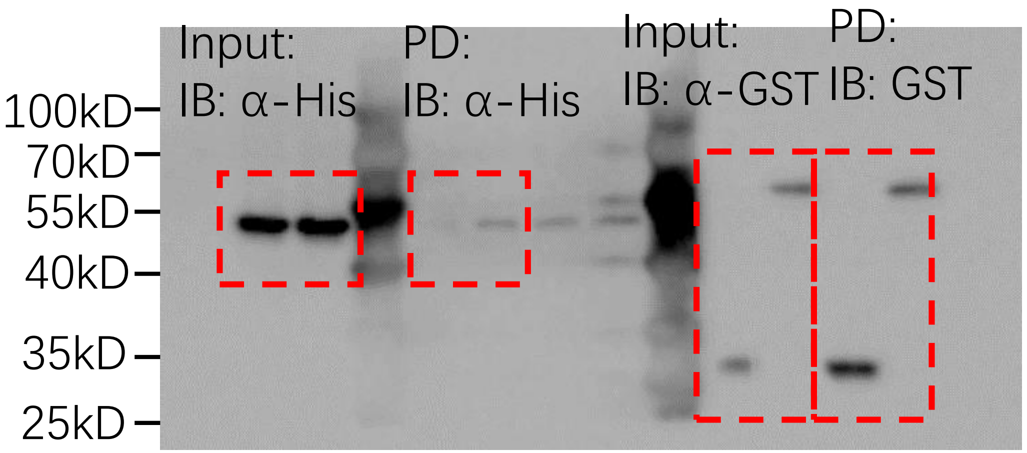

Uncropped images for gels

Figure 6g

Long exposure

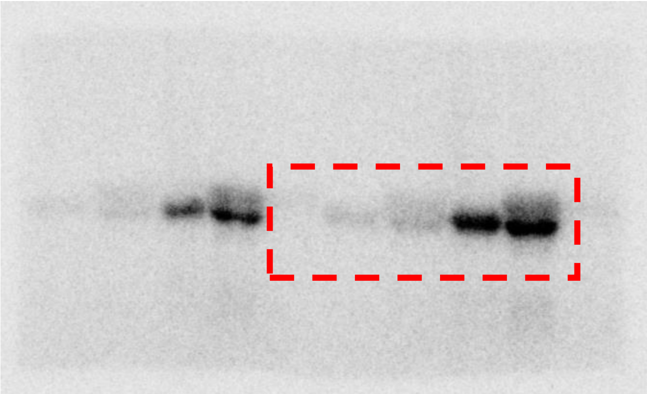

Short exposure

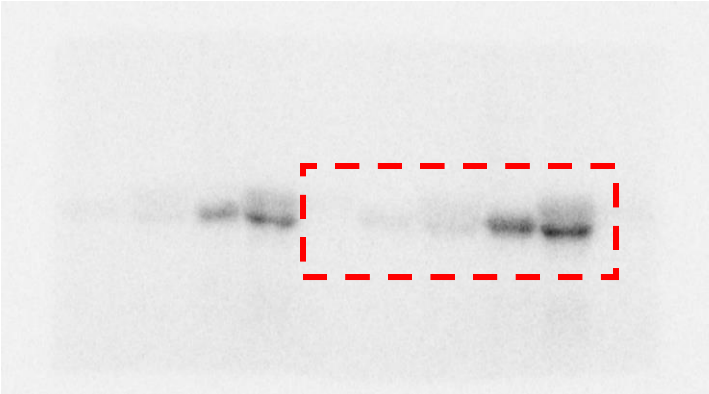

CBB

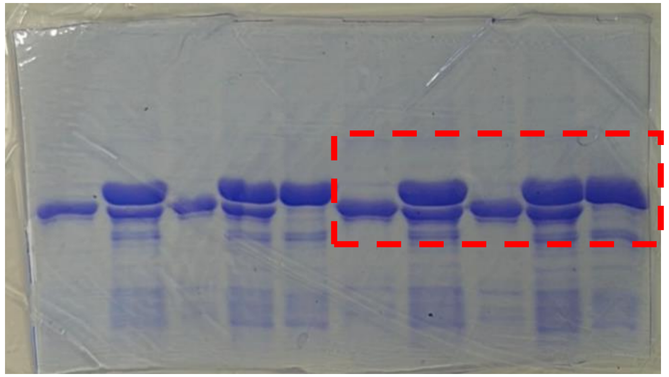

Uncropped images for gels

Figure 7b

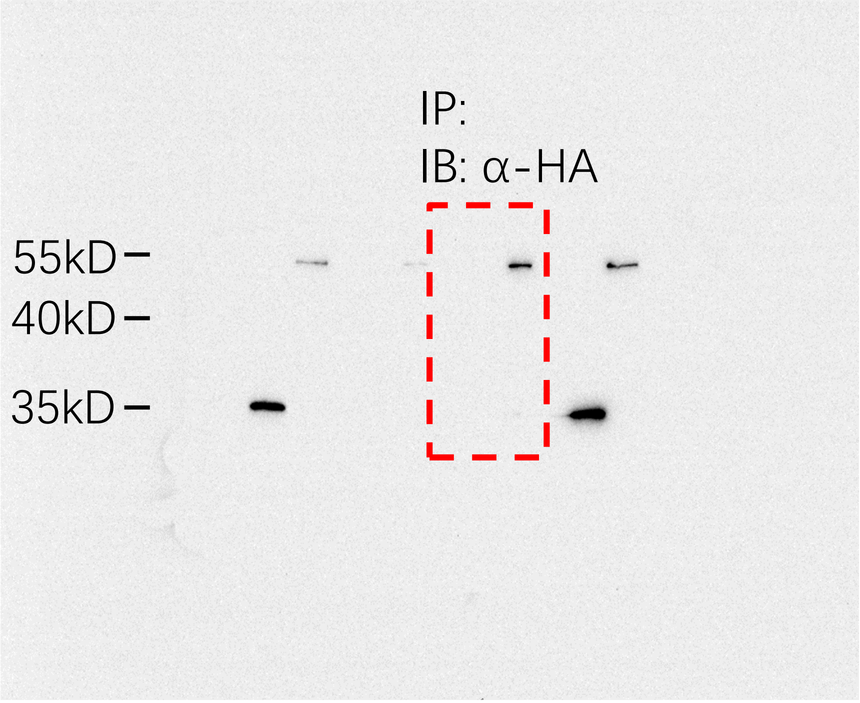

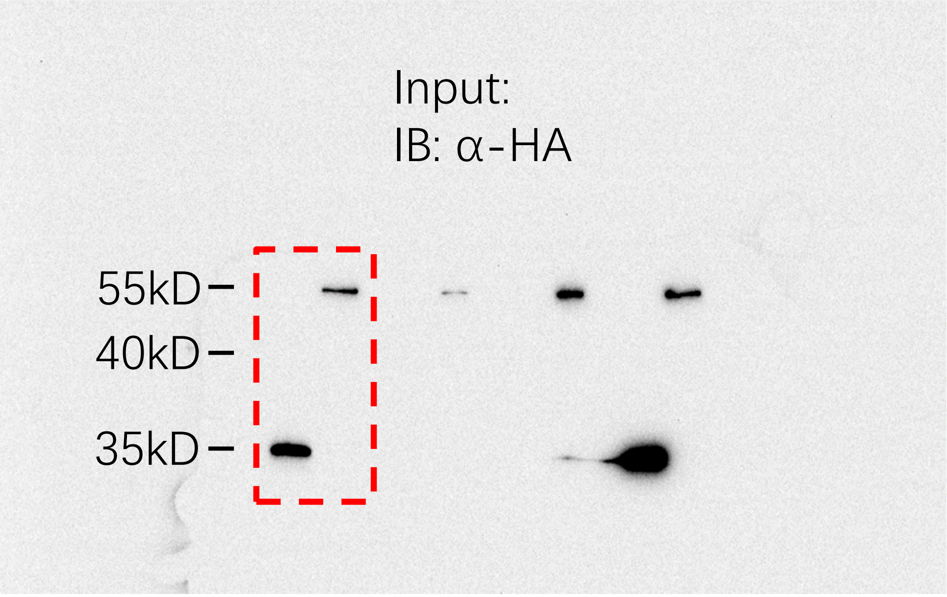

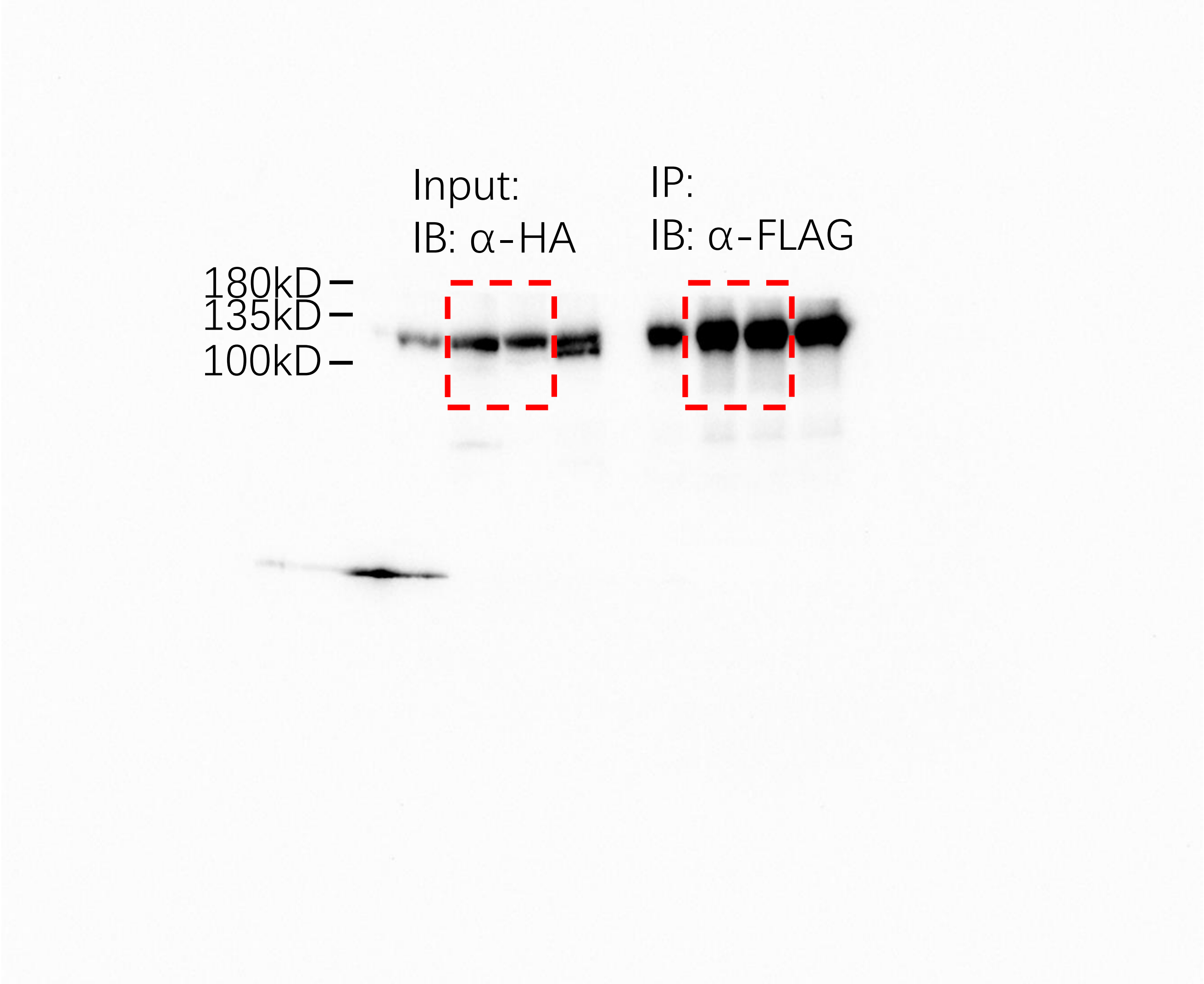

Uncropped images for gels

Figure S3

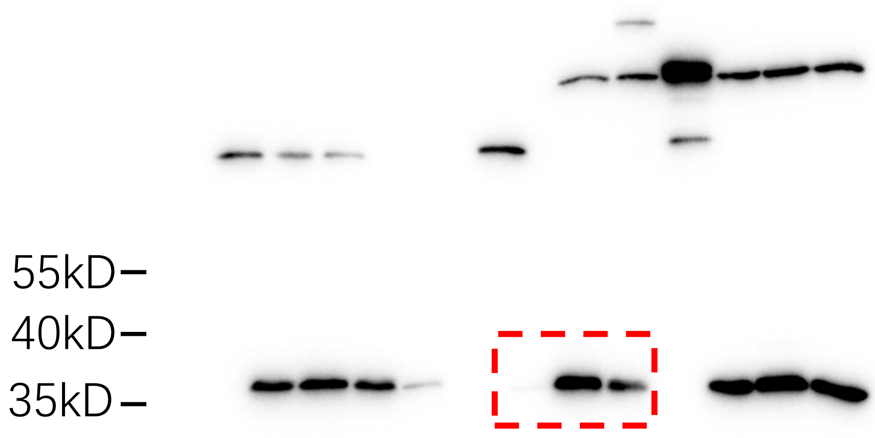

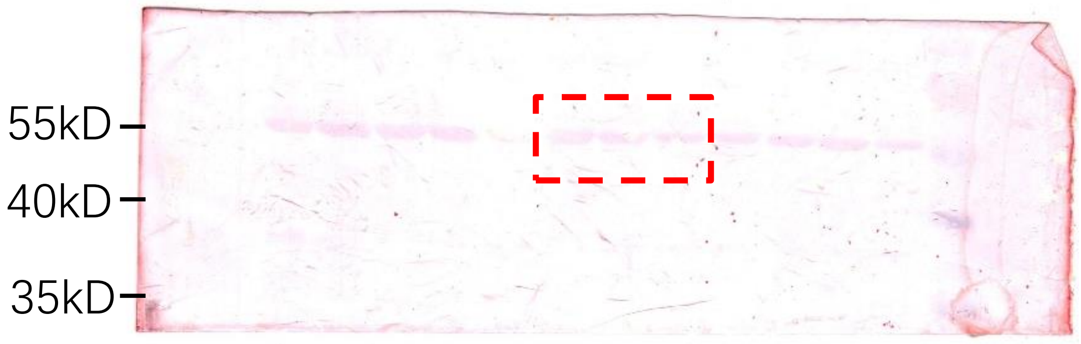

Uncropped images for gels

Figure S4e

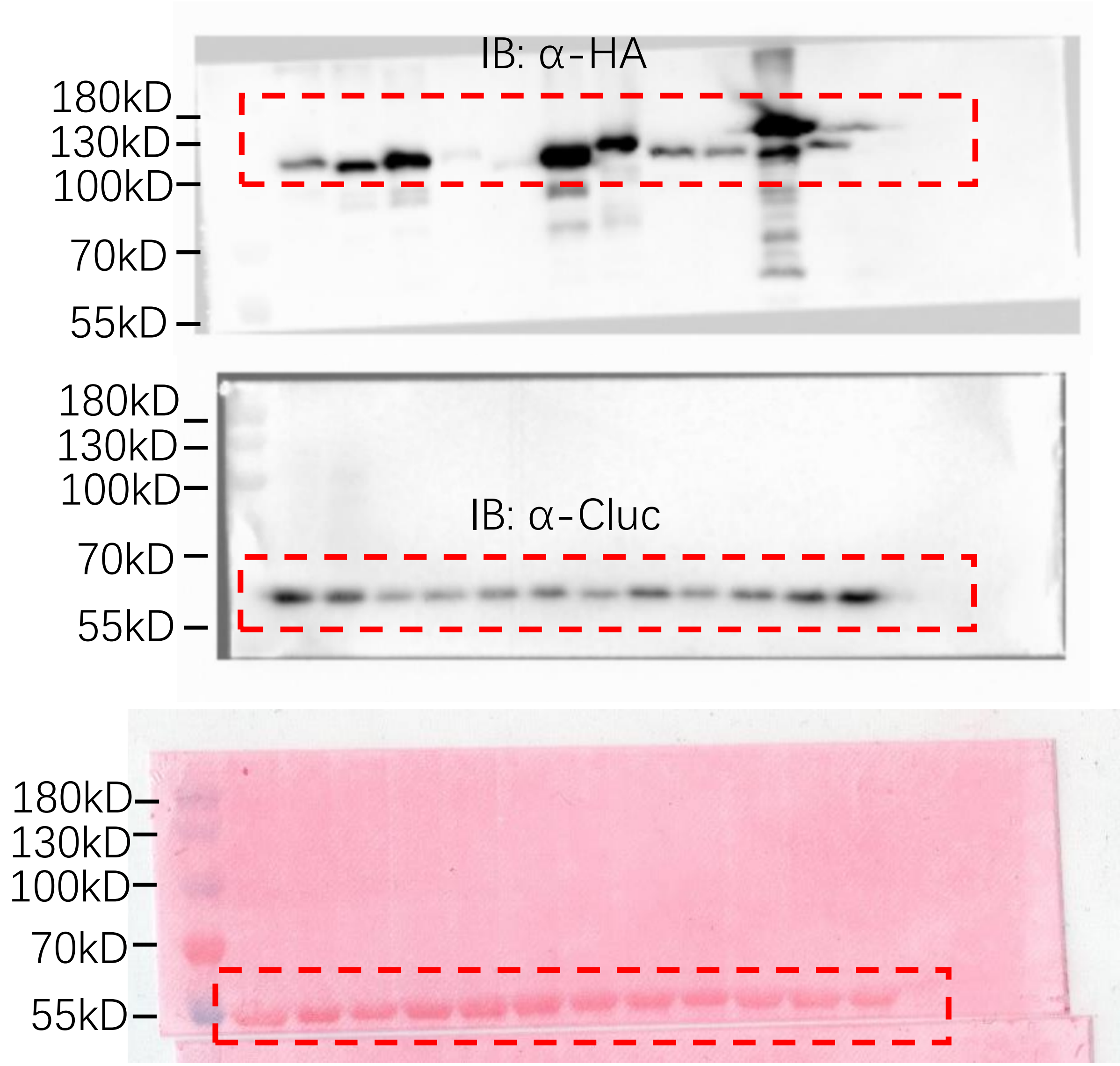

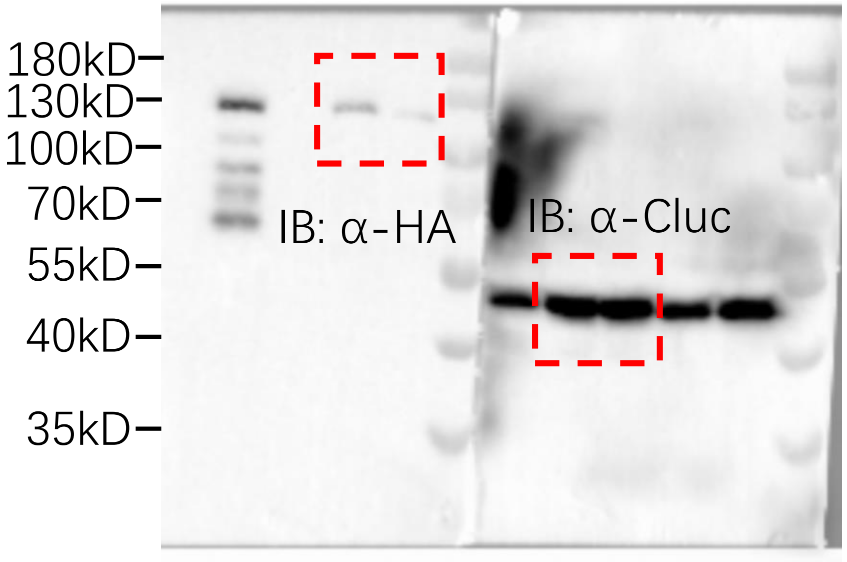

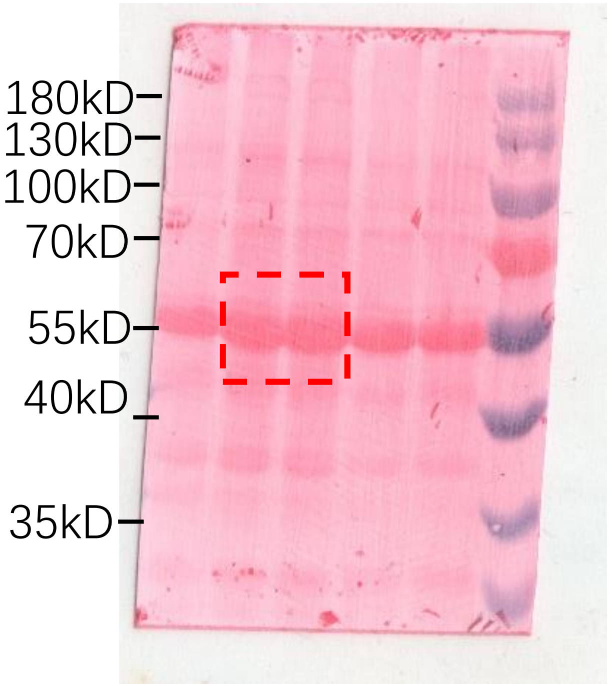

Uncropped images for gels

Figure S6b

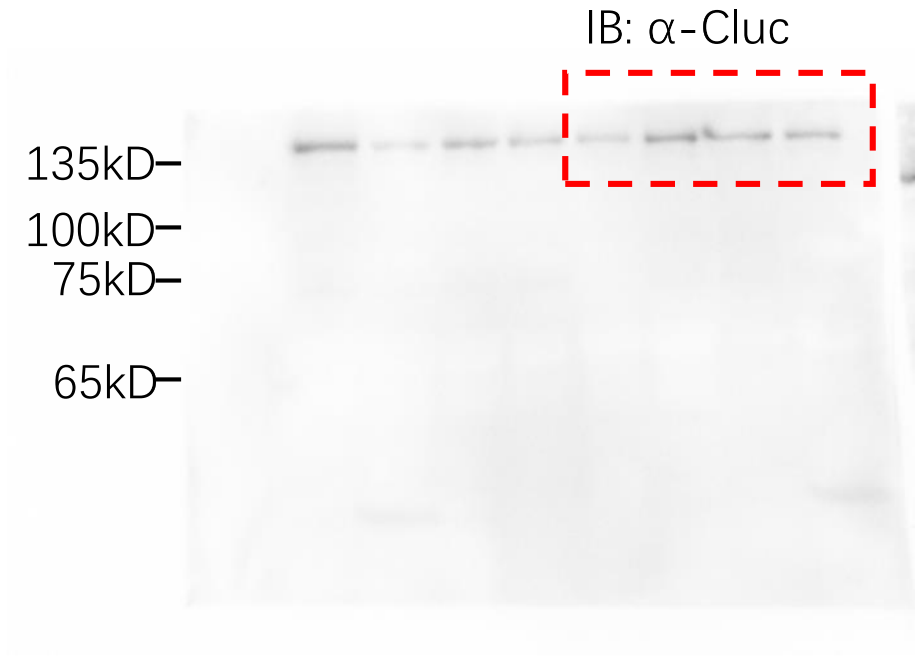

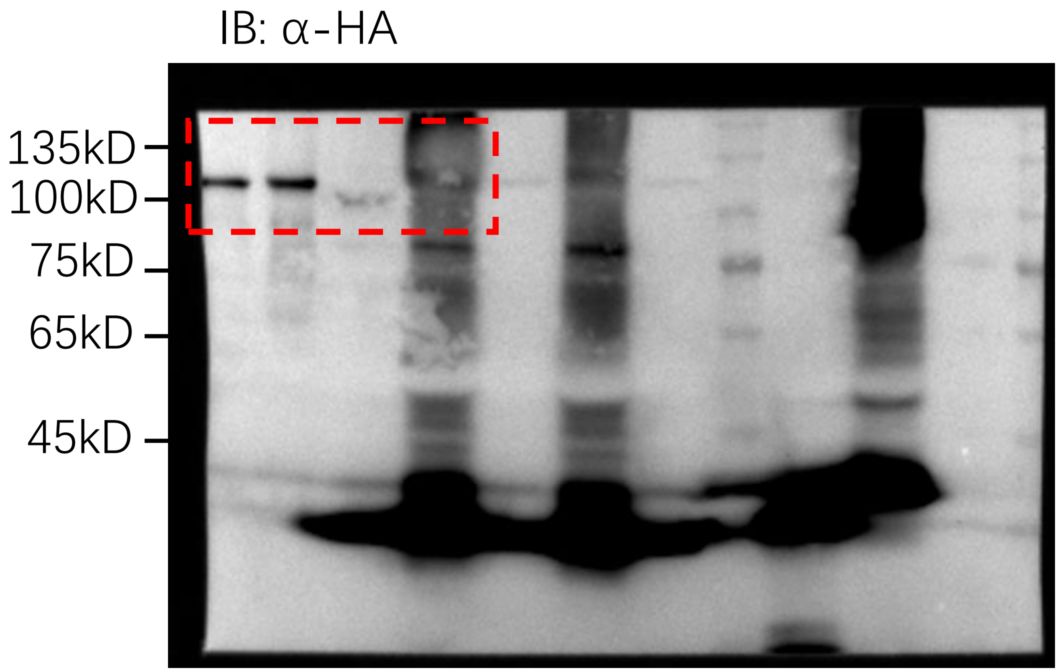

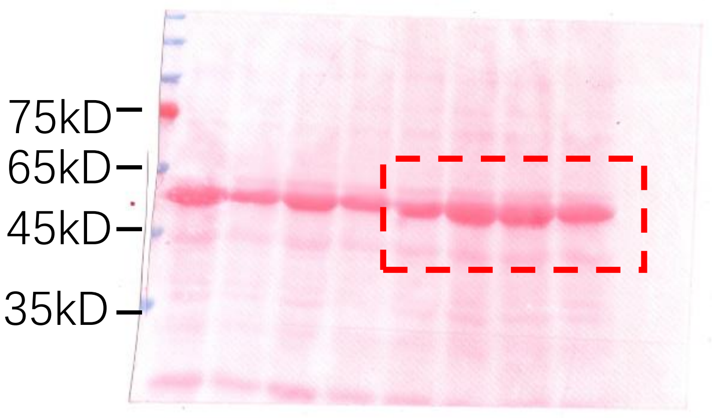

Supplement: Supplementary file 1 — Supplementary Material 1 [file 12870_2023_4713_MOESM1_ESM.pdf]
